# Unbiased cleavage site prediction uncovers viral antagonism of host innate immunity by SARS-CoV-2 3C-like protease

**DOI:** 10.1172/jci.insight.185739

**Published:** 2026-02-23

**Authors:** Nora Yucel, Silvia Marchiano, Evan Tchelepi, Germana Paterlini, Ivan A. Kuznetsov, Kristina Li, Quentin McAfee, Nehaar Nimmagadda, Andy Ren, Sam Shi, Alyssa Grogan, Aikaterini Kontrogianni-Konstantopoulos, Charles Murry, Zoltan Arany

**Affiliations:** 1 Cardiovascular Institute, Perelman School of Medicine, University of Pennsylvania, Philadelphia, Pennsylvania, USA.; 2 Institute for Stem Cell and Regenerative Medicine,; 3 Center for Cardiovascular Biology, and; 4 Department of Laboratory Medicine & Pathology, University of Washington, Seattle, Washington, USA.; 5 NetQuest Corporation, Mt. Laurel Township, New Jersey, USA.; 6 Certusoft, Bloomington, Minnesota, USA.; 7 Department of Biochemistry and Molecular Biology, University of Maryland School of Medicine, Baltimore, Maryland, USA.; 8 Division of Cardiology, Department of Medicine, University of Washington, Seattle, Washington, USA.; 9 Sana Biotechnology, Seattle, Washington, USA.; 10 Department of Bioengineering, University of Washington, Seattle, Washington, USA.

**Keywords:** COVID-19, Virology, Cardiovascular disease, Innate immunity, Proteases

## Abstract

How SARS-CoV-2 causes a wide range of clinical manifestations and disease severity remains poorly understood. SARS-CoV-2 encodes 2 proteases (3CLPro and PLPro), vital for viral production, but also promiscuous with respect to host protein targets. Pharmacological inhibition of 3CLPro markedly reduced hospitalization and death in Phase 2/3 clinical studies. Here, we develop a bioinformatic algorithm, leveraging experimental data from SARS-CoV, to predict host cleavage targets of 3CLPro. We capture targets of 3CLPro described previously for SARS-CoV-2, as well as thousands of putative targets. We validate numerous targets cleaved during infection, including the giant sarcomeric protein obscurin and the innate immune protein OAS1. A long form of OAS1, p46, has been associated in numerous GWAS studies with lesser COVID disease severity. We show that 3CLPro cleaves p46 OAS1 immediately upstream of a known prenylation domain, relocalizing OAS1 from subcellular membranes to the cytosol, rendering it akin to the nonprotective, cytosolic p42 isoform. Similar OAS1 relocalization occurs upon infection by SARS-CoV-2. Our data provide a high-throughput resource to identify putative host cleavage targets of 3CLPro and reveal a mechanism by which SARS-CoV-2 antagonizes host innate immunity in individuals with the protective p46 isoform of OAS1.

## Abstract 2

We show here that the COVID virus can cause cutting of host proteins, thereby inhibiting host immunity against the virus.

## Introduction

COVID-19 was a leading cause of death and morbidity across the world from the initial outbreak in Wuhan, China, in December 2019 until 2023 (1). How SARS-CoV-2, the causative agent of COVID-19, leads to the wide range of disease manifestations remains incompletely understood. In addition to lung damage, SARS-CoV-2 infection can also cause kidney damage, clotting disorders, loss of taste and smell, cognitive dysfunction, muscle atrophy, and cardiac dysfunction (2–5). Furthermore, long-lasting COVID-19 symptoms have been reported in patients now up to 5 years after initial illness, including fatigue, shortness of breath, brain fog, and elevated heart rate (6–8). Half a decade later, how SARS-CoV-2 affects differently the multiple organs and cell types involved, and what host characteristics determine who will develop severe or long COVID-19, remains poorly understood. A deeper mechanistic understanding of virus-host interactions is thus needed.

Various studies have identified interaction of host proteins with a number of SARS-CoV-2 components, including the spike, envelope, nucleocapsid, and membrane proteins (9–11).These interactions have various consequences, including suppression of innate immune response, apoptosis evasion, and reprogramming of host transcription and translation. In addition to protein-protein interactions, important virus-host interactions can be caused by enzymatic cleavage of host proteins by viral proteases. For example, myocardial dysfunction following infection by coxsackie CVB3 virus can in part be ascribed to cleavage of dystrophin protein by the viral protease 2A (12, 13); enteroviral 3C proteases can cleave host NLRP1 to trigger inflammasome activation (14); HIV-1 protease mediates apoptosis by cleaving host procaspase 9 and Bcl2 (15, 16); and the Zika virus nsP2 cysteine protease can cleave host proteins SFRP1, NT5M, and FOXG1 (17). Viral proteases thus often modulate pathogenic responses within the host, beyond their direct role in viral replication.

SARS-CoV-2 encodes for 2 proteases, a papain-like protease (PLPro) and the 3C-like protease (3CLPro, also knowns as main Protease, MPro, or NSP5). These proteases are highly conserved across coronavirus species and are required for viral replication (18–22). They are thus attractive targets for antiviral therapies. An early Phase 2/3 clinical trial of Paxlovid, a 3CLPro inhibitor PF-07323332 (nirmatrelvir) administered in combination with ritonavir, revealed 89% reduction in hospitalization with COVID-19 (23, 24). Paxlovid was approved for Emergency Use Authorization by the Food and Drug Administration in December 2021 for patients at high risk for progression to serious disease. The success of Paxlovid demonstrates the crucial role of 3CLPro in COVID-19 pathology.

**Figure 1:**
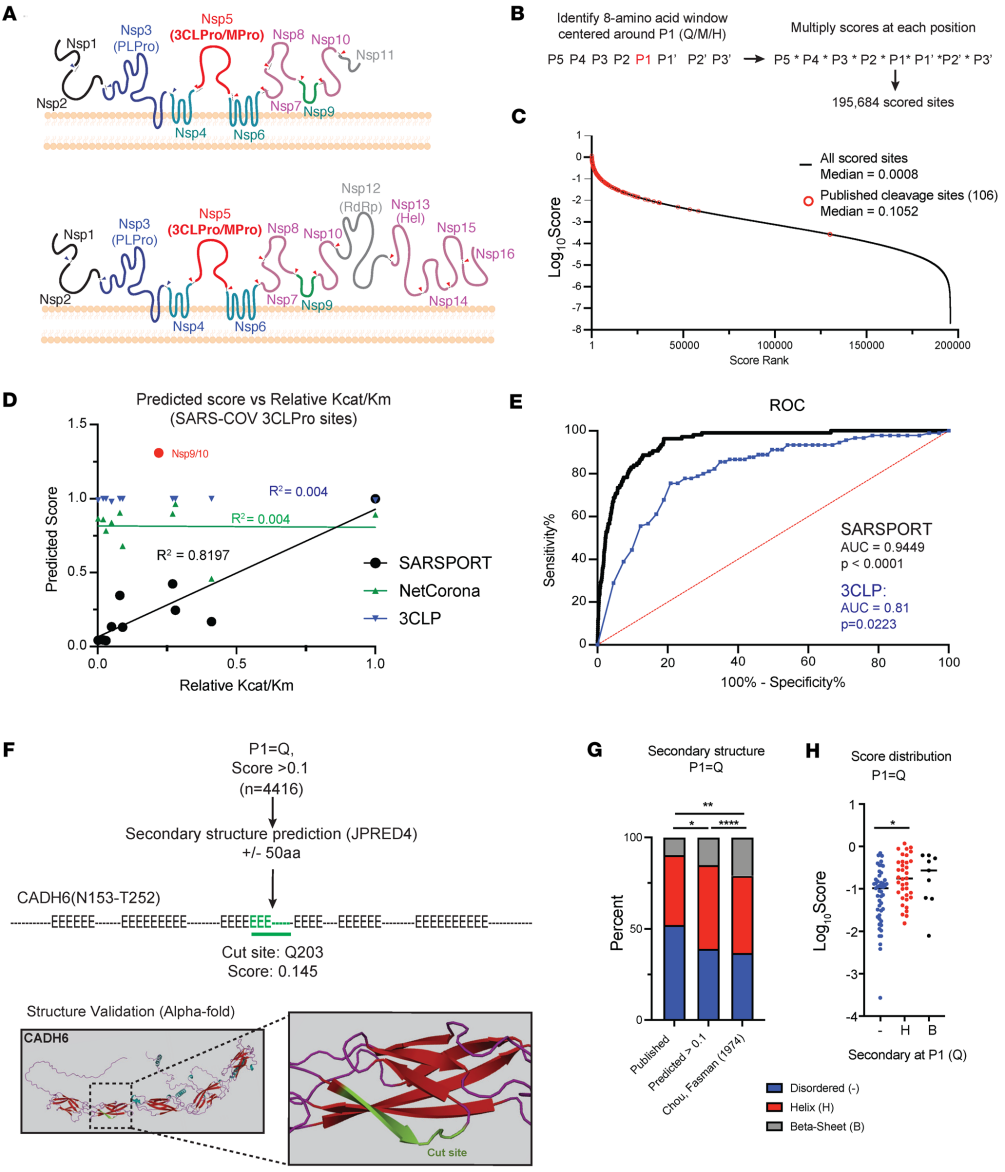
Bioinformatic prediction of SARS-COV2 3CL human protein targets. (**A**) Diagram of SARS-CoV-2 3C-like protease (3CLPro) function. 3CLPro cleaves at 11 sites within polypeptides pp1a and pp1ab, generated from overlapping reading frames ORF1a and ORF1ab. Cleavage by 3CLPro, and papain-like protease (PLPro) liberates nonstructural proteins (NSPs) required for viral function. (**B**) Identification and scoring of 3CLPro cleavage sites within the human proteome. Scores for each position along the cleavage site (P5-P3′) obtained from published SARS-CoV 2 3CLPro data. In total, 195,684 scored cleavage sites (>0) were detected across the human proteome for P1 position of M, H or Q. (**C**) Distribution of all scores (Log_10_Score). Published cleavage sites shown in red. (**D**) Correlation of predicted score with published K_cat_/K_m_ values for SARS-COV 3CLPro. Scores generated in this study compared with NetCorona1.0 and 3CLP algorithms. For R^2^ calculations, the cleavage site between NSP9 and NSP10 was excluded. (**E**) Receiver operator curve (ROC) analysis to assess predictive power of SARSPORT versus 3CLP based on scores of published cleavage sites. Cumulative percentage of scores plotted versus score rank (highest score = 1, lowest score = 100), and area under the curve (AUC) captured. A 95% CI was determined by Wilson/Brown method. (**F**) Secondary structure of high scoring sites (>0.1) with P1 = Q. Q100aa window centered around P1 was analyzed by JPRED4 to predict secondary structure (“-” = unstructured, “H” = α-helix, “E” = β-sheet). Highlighted is a predicted site in Cadherin-6 (CADH6) shown in AlphaFold. (**G**) Fraction of each P1 (Q) within each secondary structure type (unstructured, α-helix, β-sheet). Comparisons shown for predicted cleavage sites with score > 0.1 versus published cleavage sites versus published secondary structure distribution of all glutamines. Statistical analysis calculated using χ^2^ goodness of fit. (**H**) Score distribution of published cleavage sites (P1=Q) by secondary structure. Statistics calculated by 1-way ANOVA with Holm-Sidak multiple-comparison test.

Both PLPro and 3CLPro are generated via autocatalytic cleavage from the overlapping ORF1a and ORF1ab polyproteins, the first translation products following SARS-CoV-2 infection. The ORF1a/ab polyproteins encode 16 nonstructural proteins (NSP1–16) that build the viral replication machinery (Figure 1A). The nonstructural proteins are liberated from the ORF1a/ab polyprotein through cleavage by PLPro and 3CLPro, encoded by NSP3 and NSP5, respectively. Following this processing, PLPro remains bound to the endoplasmic reticulum membrane, while 3CLPro cleaves itself from the membrane into the cytosol. The effect of these proteases on the host proteome, and in particular by 3CLPro, has yet to be fully understood. Targeted screening of 300 IFN-stimulated proteins in cell lines overexpressing SARS-CoV-2 3CLPro identified the ubiquitin protein ligase BRE1A, encoded by the *RNF20* gene as a target of 3CLPro (25). In a different screening of 71 immune pathway-related proteins, the IFN regulatory transcription factor IRF3 was identified as a target of PLpro, and NLRP12 and TAB1 as targets of 3CLPro, suggesting the role of those proteases in the innate immune response to the virus (26).

Only limited efforts have thus far been taken to identify systematically, and in an unbiased fashion, host cleavage targets of 3CLPro from SARS-CoV-2. Given the high conservation of 3CLPro, such analysis would also extend across other coronavirus species. One approach taken used N-terminomics to identify neo-N-termini generated by the viral proteases; 14 new cellular (27) and > 100 substrates (28) were identified in 2 different global studies in either SARS-CoV-2–infected cell lines, or 3CLPro-treated cell lysates. This approach is limited, however, by the need for sufficient protein abundance and appropriate fragment size to be detected by mass spectrometry (29, 30). Comparing these published targets, only 3 protein targets have been identified by more than 1 study (TAB1, ATAD2, and NUP107). The lack of overlap across different studies reflects the lack of saturation of these experimental approaches. Moreover, cleaved proteins that are subsequently degraded (a process accelerated by infection) (31, 32) also escape detection by N-terminomic approaches. In silico approaches provide the opportunity to overcome these limitations and to avoid laborious experimental screens. An initial such approach relied on similarity between cleavage sites in the viral polypeptide across divergent human and nonhuman coronavirus species (NetCorona1.0) (33) to predict SARS-CoV 3CLPro targets. However, this method often does not match experimental cleavage studies on SARS-CoV, likely because of the divergent coronavirus species used for identifying targets and generating scores. For example, NetCorona1.0 predicts that a sequence containing a proline at the P2′ position can be cleaved, but this substitution has been shown to block cleavage in cleavage assays (34). In addition, NetCorona1.0 does not consider cleavage site accessibility conferred by secondary structure, the relative efficiency of cleavage at different sites, or the possibility that there may be host target sites of higher affinity than viral sites. Nevertheless, these in silico methods and screens have uncovered several validated host targets of 3CLPro, from proteins involved in transcription and translational machinery (28, 35–37) to antiviral and immune signaling pathways (26, 38–42). The wide scope of validated targets underscores the critical role of 3CLPro in mediating disease pathology and highlights the need for improved methods for prediction of host protein cleavage by 3CLPro.

Here we combine published cleavage efficiency data on the SARS-CoV 3CLPro, which is 96% similar to SARS-CoV-2 3CLPro (43), with genome-wide secondary structure analyses, to identify and score 99,000+ predicted SARS-CoV/SARS-CoV-2 3CLPro cleavage sites across the human proteome. Through score filtration and secondary structure analysis, we identify over 1,000 high-likelihood sites. We rediscover nearly all prior SARS-CoV-2 3CLPro experimentally identified sites, and we experimentally validate newly identified prominent targets with purified reagents and in cell culture. We tested cleavage targets in infected cardiomyocytes (CM) as proof-of-concept validation of targets that are large structural proteins, whose size would make them more likely to possess cleavage sites. We show that 3CLPro expression leads to cleavage and degradation of the giant sarcomeric protein obscurin in human induced pluripotent cell–derived CMs (hiPSC-CM) and recapitulates the sarcomeric disorganization observed with SARS-CoV-2 infection in hiPSC-CMs (38, 44, 45). In addition, we demonstrate degradation of obscurin in SARS-CoV-2–infected hPSC-CMs. We further use comparative bioinformatic analyses with identified loci in genome-wide association studies (GWAS) to identify the innate immune defense protein OAS1 as a predicted 3CLPro target, and we show that 3CLPro cleaves OAS1 directly, leading to its release from intracellular membranes, its primary site of action. Our study provides a comprehensive atlas for identifying the degradome of 3CL proteases, applicable to SARS-CoV-2 and, in light of the structural conservation of the 3CL protease, across coronavirus species (46) in possible future coronavirus outbreaks. Our study also reveals a mechanism by which SARS-CoV-2 antagonizes host innate immunity.

## Results

### Bioinformatic prediction of SARS-CoV-2 3CLPro targets using experimental data from SARS-CoV 3CLPro.

We first sought to identify and score potential cleavage targets of the 3CLPro encoded by SARS-CoV2. Given the 96% sequence similarity between 3CLPro from SARS-CoV-2 and SARS-CoV as well as the homology in the viral genome cut sites (18, 25, 46), we developed an algorithm based on experimental data generated previously from SARS-CoV (2003) 3CLPro (33). In this previous study, FRET polypeptides spanning the first endogenous cut site between NSP4 and NSP5 (P5-SAVLQSGF-P3′) were generated and modified with every possible single amino acid substitution from P5 to P3′ position relative to the cleavage site. Cleavage efficiency by 3CLPro was then assessed by fluorescence intensity compared with the consensus cleavage sequence. We leveraged this data set to generate a score for every possible cleavage site using a lookup table, multiplying the relative efficiency of each amino acid. This multiplication was then applied with a sliding 8-amino acid windows across the entire human proteome (Figure 1B). Substitution at any site that showed no detectable cleavage was interpreted as “0.” Assuming a glutamine (Q) in the P1 position, over 98,697 scored sites (>0) were identified. Expanding our search to include methionine (M) or histidine (H) at P1 uncovered a total of 195,684 sites with a median score of 0.0008 (Figure 1C and Supplemental Table 1; supplemental material available online with this article; https://doi.org/10.1172/jci.insight.185739DS1). GO analysis of scores in the top 15% (>0.01) showed an enrichment for cell adhesion, morphogenesis, and cytoskeletal genes (Supplemental Table 2). We named the algorithm Sarsport1.0.

To evaluate the accuracy of Sarsport1.0, we calculated scores for the 11 known 3CLPro cut sites in the SARS-CoV viral genome. Scores ranged from 1.31 to 0.04, all within the upper fifth percentile of the score range. These scores were then compared with the published relative K_cat_/K_m_ values for each cleavage site (47). With the exception of the cut-site between NSP9 and NSP10 (ATVRLQ*AGNAT), our calculated scores correlated closely with the relative K_cat_/K_m_ determined for the remaining 10 known 3CLPro cleavage sites. In contrast, there was essentially no correlation between K_cat_/K_m_ and cleavage scores generated by either NetCorona1.0 or a recently reported machine-learning algorithm, 3CLP (Figure 1D) (39).

To evaluate the sensitivity of Sarsport1.0 to identify SARS-CoV-2 host protein targets, we next calculated the scores for the > 100 SARS-CoV-2 3CLPro cleavage targets recently identified via N-terminomics and screening approaches (26–28). Sarsport1.0 identified 106 of the 122 cleavage sites, including those with noncanonical methionine or histidine at the P1 position (Figure 1C). The median score was over 0.1, which is within the top 2.5% of all scores. Receiver operator curve (ROC) analysis (Figure 1E) showed Sarsport1.0 to be highly predictive, with an AUC of 0.9449 and *P* < 0.0001. In comparison, 3CLP identified only 90 sites, in large part due to exclusion of sites with methionine or histidine at the P1 position. While experimentally identified sites were enriched for higher scores (0.97 for experimental sites versus 0.73 for all identified 3CLP sites), 3CLP scores were not as predictive as Sarsport1.0. Receiver operator analysis showed AUC of 0.81 and *P* value of 0.02. We surmise this is likely due to the fact that 3CLP, like NetCorona1.0, generates likelihood scores based on evolutionary homology to other coronavirus sites, versus relative efficiency of the amino acids at each position. We conclude that Sarsport1.0 is highly predictive of cleavage sites by both SARS-CoV and SARS-CoV-2 3CLPro proteases.

### Refinement of cleavage prediction by secondary structure analysis.

The unique high score but low K_cat_/K_m_ of the NSP9/10 cleavage site (Figure 1D) suggested that a higher order structure, not captured by scoring based on primary sequence alone, might inhibit cleavage. We therefore estimated the secondary structure of each cut site in the viral genome, using the JPRED4 protein secondary structure prediction server (48) and a 100 amino acid (aa) window spanning the P1 position. The NSP9/10 site in SARS-CoV was the only cleavage site where the P1 position (Q) was predicted to lie in a β-sheet (Supplemental Table 3). In contrast, the other sites all lay in predicted α-helices or disordered regions, structures known to be more accessible to proteases (49, 50). These data suggest that higher order structures such as β-sheets hinder cleavage by 3CLPro.

To further probe this possibility, we used JPRED4 to evaluate secondary structures around all predicted cleavage sites with a Q at P1 and with a Sarsport1.0 score > 0.1, adding up to 4,416 sites (Figure 1F and Supplemental Table 4). The recent publication of predicted structures for most of the human proteome with AlphaFold (51) also provides the opportunity to cross-validate secondary with higher order structures. The relative frequency of β-sheet structures at the P1 position of predicted cleavage sites was significantly lower than the overall frequency of β-sheets for Qs in the proteome (52) (Figure 1G), indicating that Sarsport1.0 partly biases away from β-sheets. Secondary analysis of experimentally identified, published cleavage sites revealed an additional significantly increased propensity for cleavage in regions where P1 (Q) is unstructured and in particular not in a β-sheet (Figure 1G). Thus, filtering results from Sarsport1.0 for the absence of a β-sheet structure at P1 will improve its positive predictive value. Interestingly, the median Sarsport1.0 score for sites that lie in unstructured regions (0.1024) was significantly lower than for sites that lie in α-helices (0.1727) or β-sheets (0.27) (Figure 1H), suggesting that the presence of a less permissive secondary structural order imposes a higher evolutionary pressure for an optimal primary sequence cleavage motif.

### Cleavage validation of targets.

**Figure 2:**
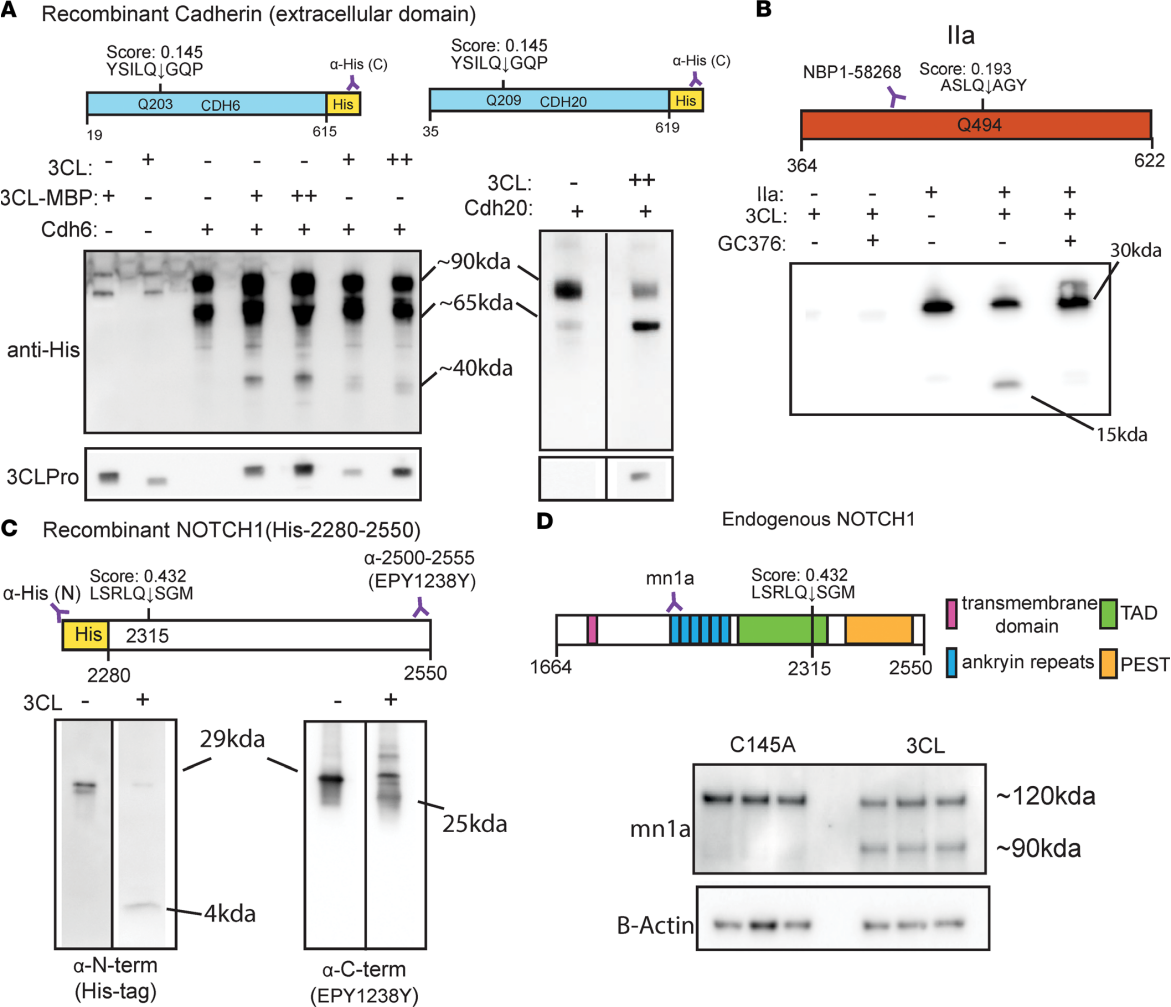
Validation of predicted protein targets. (**A**) Western blot of in vitro cleavage of recombinant cadherin with 3CLPro. Shown are cleavage sites within the recombinant fragment, amino acid positions displayed for the full length proteins. Western blots show staining against the C-terminus (His-Tag) of each protein and 3CLPro. Recombinant proteins are a mixture of glycosylated (~90 kDa) and unglycosylated (~65 kda), corresponding to cleavage fragments of ~62 kDa and 40 kDa (respectively). For CADH6 cleavage, 2 formulations of 3CLPro were tested (3CLPro unconjugated, 3CLPro-Maltose-Binding Protein conjugated) at 2 concentrations of protease (+, 0.5 μM; ++, 1 μM). For CADH20 cleavage, only unconjugated 3CLPro digests at 1 μM concentration are shown. For 3CLPro and CADH20 staining (His-Tag), samples were run on the same gel but are noncontiguous (as indicated by line separating lanes). (**B**) Western blot of in vitro cleavage of purified human α-thrombin (IIa). Diagram shows amino acid position of unprocessed prothrombin. Position of cleavage site shown with respect to epitope of antibody used for Western blot. In total, 1 μM of purified 3CLPro was incubated with 2 μg α-thrombin overnight under reducing conditions, with or without the 3CLPro inhibitor GC376 (1 μM). (**C**) In vitro cleavage of purified recombinant NOTCH1 fragment (aa 2280–2550) with a N-terminal His-Tag. Reactions were done with 1 μM of purified 3CLPro for 1 hour. Diagram shows position of cleavage within the NOTCH1 fragment, with amino acid positions corresponding to the full-length protein. Epitope regions showed for antibody with epitope C-terminal to the cleavage site. Full-length size is ~29 kDa, with N and C-terminal fragments of 4 kDa and 25 kDa (respectively). Samples were run on the same gel but are noncontiguous. (**D**) Cellular cleavage of NOTCH1. Western blots show lysates of hIPSC cardiomyocytes expressing 3CLPro or catalytically inactive C145A variant for 48 hours. Cleavage site position within the intracellular fragment of NOTCH1 shown, as well as epitope for antibody used in Western blot.

Because of the higher sensitivity of our method, we identified numerous new predicted cleavage sites, in addition to those previously published. Gene Ontology (GO) analysis of proteins with Sarsport1.0 score > 0.01 showed enrichment for cytoskeletal, cell motility, and cell adhesion proteins, including several predicted cleavage sites located within homologous cadherin domains in the cadherin protein superfamily (Supplemental Table 2). Evaluation with AlphaFold predicted these sites to be in unstructured accessible loops within the cadherin domain, thus making them likely to be cleaved if exposed to 3CLPro (Figure 1, G and H). We validated these hits in vitro by incubation of purified 3CLPro with commercially available recombinant cadherin proteins (CDH6, CDH20), which have identical predicted sites (Score 0.145; Q203 and Q209, respectively; Figure 2A). 3CLPro efficiently cleaved both CDH6 and CDH20, yielding the expected fragment sizes based on the predicted cleavage site (Figure 2A). We similarly validated cleavage sites in thrombin (IIA) and the intracellular domain of NOTCH1 as in vitro reactions with both purified proteins yielded the expected fragment sizes (Figure 2, B–D). The appearance of the thrombin IIA cleaved product was inhibited by the 3CLPro inhibitor GC376, demonstrating the requirement of 3CLPro enzymatic activity (Figure 2B). Moreover, cleavage of NOTCH1 at a predicted site (Q2315, score 0.432), located within its intracellular domain, yielded both predicted fragments (Figure 2C). Expression of 3CLPro versus the catalytically inactive C145A mutant in hiPSC-CMs also yielded NOTCH1 fragments of the predicted length, demonstrating cleavage in intact cells (Figure 2D). Additional targets chosen for their high score and secondary structure accessibility (i.e., SVIL, UACA, NOTCH2) were similarly validated with 3CLPro expression in 293T cells, as was the previously published target TAB1 (Supplemental Figure 1A). In a number of these studies, levels of full-length target proteins were reduced by expression of 3CLPro, but the detection of fragments was not always observed, suggesting that cytosolic fragments generated by 3CLPro may be further degraded by endogenous proteolytic pathways. Supporting this notion, the plasma-membrane bound N-terminal cleavage product of full length NOTCH1 yielded the expected 90 kDa fragment, while the C-terminal cleavage site only showed reduction in total protein levels (Supplemental Figure 1B). We conclude that 3CLPro can cleave a wide range of host proteins and that the generated cytosolic protein fragments are likely often degraded by endogenous pathways.

### Cardiac targets of SARS-CoV-2 3CLPro show multiple cut sites across sarcomeric proteins.

**Figure 3:**
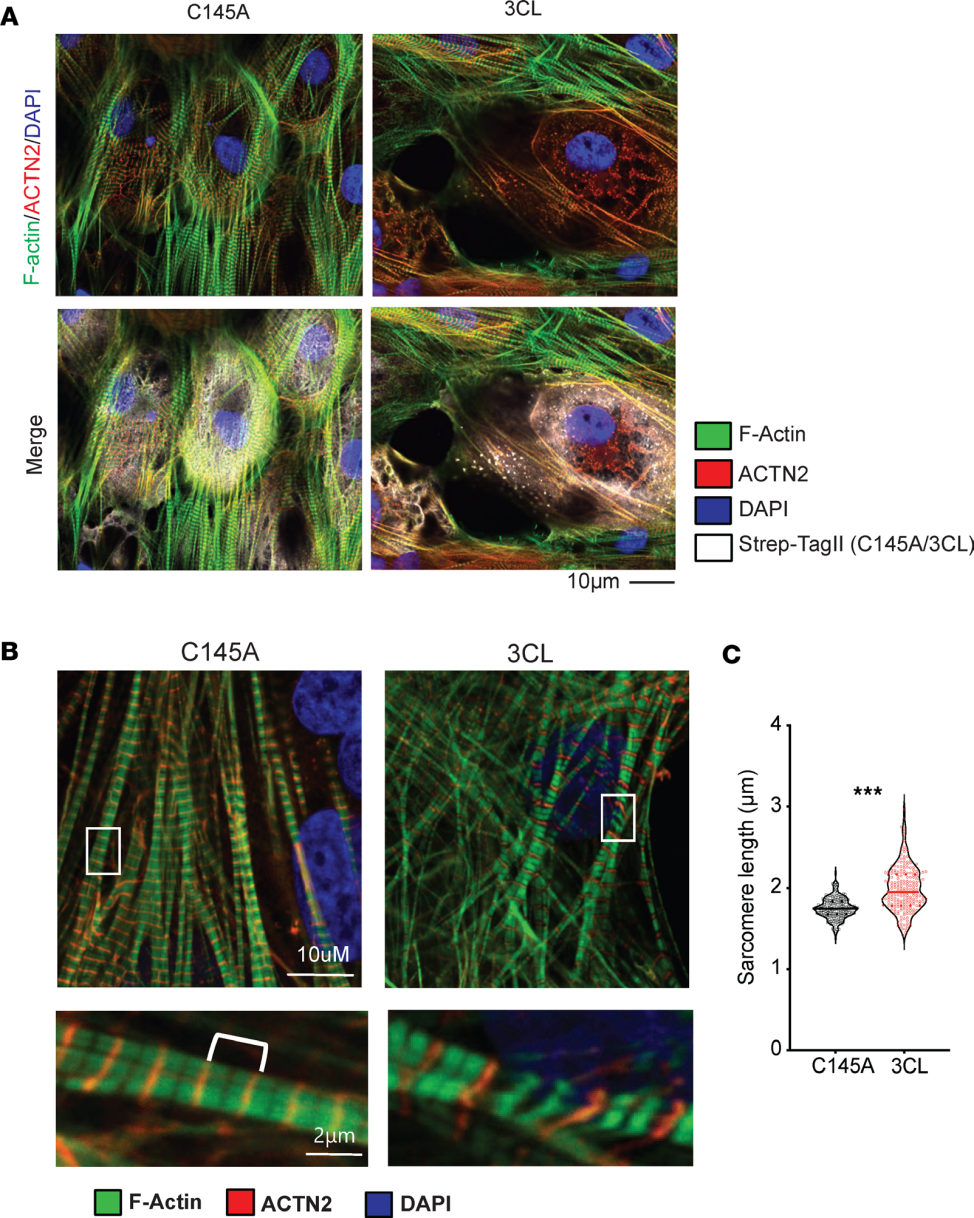
Sarcomere breakdown with 3CLPro expression. (**A**) Sarcomere breakdown with overexpression of 3CLPro. hiPSC-CMs transduced with adenovirus-overexpressing Strep-Tagged 3CLPro or catalytically inactive C145A control for 48 hours. Staining shows α-actinin (ACTN2), F-actin (phalloidin), and Strep-Tag. DAPI counterstain. Scale bar: 10 μm. (**B**) Increased sarcomere length with overexpression of 3CLPro. Sarcomeres are stained F-actin (Phalloidin) and ACTN2 to mark Z-disks. Upper panel shows representative images of cardiomyocytes, while bottom panel shows zoomed in images of representative normal and extended sarcomere distances. Scale bars: 10 μm (upper), 2 μm (bottom). (**C**) Quantification of sarcomere distance. Sarcomere lengths (Z-disk to Z-disk length, as stained by ACTN2) were quantified for ~200 sarcomeres measured for 10–15 cells each for *n* = 2 independent experiments (>400 sarcomeres total). Statistics shown by Student’s unpaired 2-tailed *t* test (***P < 0.001).

Previous work has demonstrated disorganization of sarcomeres after SARS-CoV-2 infection of hPSC-CMs (38, 44, 45, 53). We hypothesized that 3CLPro may be degrading sarcomere proteins directly. Consistent with this notion, expression of 3CLPro, but not a catalytically inactive mutant (C145A), in hiPSC-CMs led to pronounced sarcomere breakdown within 48 hours (Figure 3A). At this 48-hour time point, we also observed numerous cells with a stereotypical intermediate phenotype, in which sarcomeres exhibited increased length, as defined by the distance between α-actinin stained Z-discs (Figure 3, B and C), suggesting that key structural protein(s) of the sarcomere may be targeted by 3CLPro.

**Figure 4:**
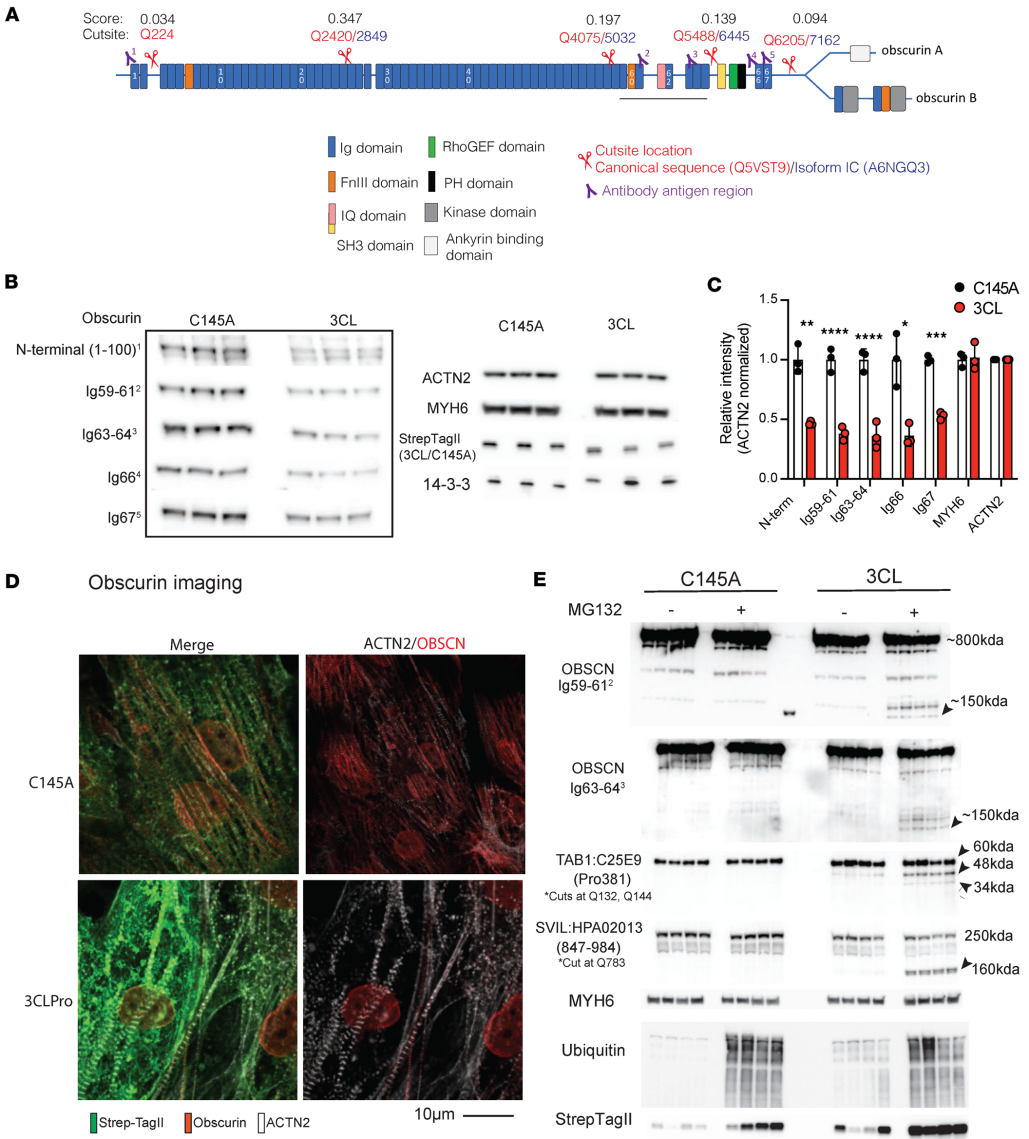
Obscurin cleavage with 3CLPro expression. (**A**) Schematic of Obscurin domain structure (generated from accession #NP_001258152.2/Unitprot ID A6NGQ3) with overlayed predicted cleavage sites (based on “canonical” UniProt Q5VST9 sequence) and predicted ~150 kDa fragment arising from Q4075 and Q5488 cleavage events. In red are cut-site locations based on the canonical sequence Q5VST9; in blue are the locations based on A6NGQ3. Epitope regions for antibodies used for this study are labeled 1–5. (**B**) Obscurin expression after 72 hours of 3CLPro or catalytically inactive C145A. Western blots are shown for all 5 obscurin antibodies, as well as ACTN2 and MYH6 as sarcomeric protein controls run on tris-acetate gels. 14-3-3 shown as loading control, as well as StrepTagII to mark 3CL/C145A expression, with proteins run separately on standard tris-glycine based gels. (**C**) Quantification of blots shown in **B**, normalized by ACTN2 staining run for each obscurin blot on the same membrane. Significance determined by Ordinary 2-way ANOVA with Šidák’s multiple-comparison test. *N* = 3 biological replicates. (**D**) Immunocytochemistry for obscurin and ACTN2 after 48 hours overexpression of 3CLPro or catalytically inactive C145A. Scale bar: 10 μm. (**E**) Expression of cleavage targets following expression of 3CLPro or catalytically inactive C145A. Following 24 hours of overexpression, MG132 (1 μM) or vehicle was added for 24 hours for a total of 48 hours overexpression with or without MG132. Fragments are highlighted for OBSCN, TAB1, and SVIL. Location of the obscurin fragment is underlined in black in the schematic in **A**. MYH6 shown as an loading control, Ubiquitin shown to demonstrate efficacy of MG132 addition, and StrepTagII staining shown to indicate 3CL or C145A expression.

We applied our in silico primary and secondary analysis to identify putative sarcomeric targets of 3CLPro(Supplemental Table 5). Within this list, we identified the giant cytoskeletal protein obscurin encoded by the *OBSCN* gene, as a probable target, with 5 high-likelihood sites (i.e., Q224, Q2420, Q4075, Q5488, Q6205) that span the length of both giant isoforms, obscurin A (~720 kDa) and B (~870 kDa) (Figure 4A). Consistent with these data, hiPSC-CMs expressing 3CLPro had a marked reduction in obscurin protein levels compared with cells expressing the C145A mutant, which was apparent using antibodies against 5 different epitopes along the length of the protein, from the N-terminal immunoglobulin domain 1, obscn-Ig1, to the C-terminal obscn-Ig67 (Figure 4, B and C). In contrast, the levels of α-actinin (encoded by *ACTN2*) and myosin heavy chain 6 (encoded by *MYH6*), which our algorithm did not predict to have cleavage 3CLPro sites in structurally accessible regions, were not altered (Figure 4, B and C). These findings were further corroborated by immunocytochemistry, which showed reduced obscurin presence in otherwise apparently intact, α-actinin^+^ sarcomeres in hiPSCs expressing 3CLPro, but not inactive C145A (Figure 4D).

As with a number of targets described above (Supplemental Figure 1), we did not detect any obscurin fragments despite using antibodies that recognize multiple epitopes along the entire length of the protein. To test whether the absence of fragments might be due to endogenous proteosome activity, we treated CMs expressing 3CLPro, versus C145A, with the proteosome inhibitor MG132, and we collected cellular proteins 24 hours later. Blotting with antibodies to obscn-Ig59/61 and obscn-Ig63/64, which recognize the region between cut sites 3 and 4 (Q4075 and Q5488, respectively), yielded the expected fragment size (~150 kDa; Figure 4E), validating the predicted sites as a 3CLPro targets, and demonstrating that the ensuing fragment is targeted for degradation by the proteosome. Identification of other fragments within obscurin was technically unfeasible due to either lack of antibodies against the specific region or due to comigration with nonspecific immunoreactive bands or smaller endogenous obscurin isoforms (54). Notably, proteosome inhibition also uncovered fragments from other high-scoring targets, including Supervillin (SVIL), another giant sarcomeric protein predicted to be targeted by our algorithm, as well as TAB1, which was previously shown to be targeted by 3CLPro but for which no fragments had been detected (Figure 4E) (26).

### Obscurin degradation in SARS-CoV-2 infection.

**Figure 5:**
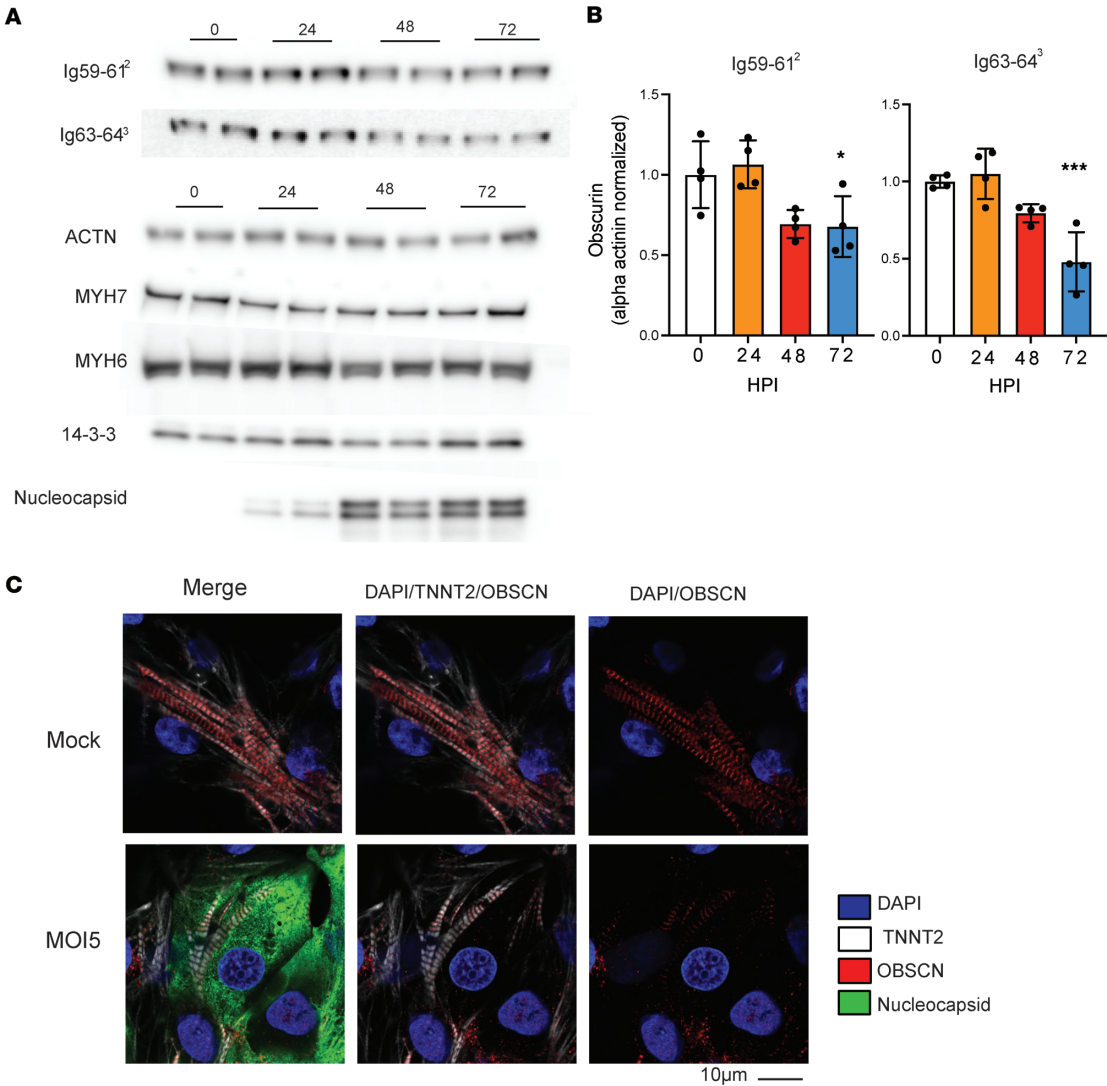
Obscurin degradation in SARS-COV2 infection. (**A**) Western blots of WTC-11c hPSC-CMs infected with SARS-CoV-2 at 5 MOI after 24, 48, and 72 hours. (**B**) Quantification of obscurin and myosin heavy chain 6 and 7. Intensity normalized first for ACTN2 (per blot), then normalized for T0 values of each cell type. Shown are *n* = 2 for each time point for 2 different hPSC-CMs cell lines (WTC-11c and H7) for a total of *n* = 4. Significance of normalized obscurin intensity compared with time 0 was calculated by 1-way ANOVA with Dunnett’s test for multiple comparisons. (**C**) Immunocytochemistry for obscurin in WTC-11c hPSC-CMs at 48 hours post-infection (HPI) with MOI 5 SARS-CoV-2. TNNT2 used as a counterstain for sarcomeres, and nucleocapsid staining performed to identify infected cells. Panels (left to right) show representative merged image, DAPI/TNNT/OBSCN overlay to emphasize sarcomeres, and DAPI/OBSCN only to highlight loss of OBSCN. Scale bar: 10 μm.

We next tested if those results were recapitulated in a model of human pluripotent stem cell cardiomyocytes (hPSC-CMs) infected with live SARS-CoV-2. For these experiments, we used 2 hPSC-CM lines, WTC-11c (hiPSC-CMs) and H7 (human embryonic stem cell–derived CMs [hESC-CMs]), previously used to study the effects of SARS-CoV-2 on human CMs (45). Within 48 hours of infection, coincident with the robust appearance of viral nucleocapsid, total obscurin protein gradually reduced by 40%–60% (Figure 5, A and B). In contrast, protein abundance of ACTN2, MYH6, and MYH7 was unaffected by infection (Figure 5, A and B), mirroring the effects seen with 3CLPro alone (Figure 4, B and C). Similarly, immunocytochemistry of infected cells showed reduction of obscurin staining in cells expressing viral nucleocapsid, despite seemingly intact sarcomeres, determined with troponin T staining (Figure 5C), again mirroring the effects seen with 3CLPro alone (Figure 4D). Thus, the loss of obscurin caused by 3CLPro-mediated cleavage might explain the loss of sarcomere integrity during SARS-CoV-2 infection in human CMs, consistent with its key role in sarcomeric organization and pathological remodeling (55–58).

### Colocalization with loci associated by GWAS with disease severity identifies a disease-relevant cleavage site in the innate immunity protein OAS1.

**Figure 6:**
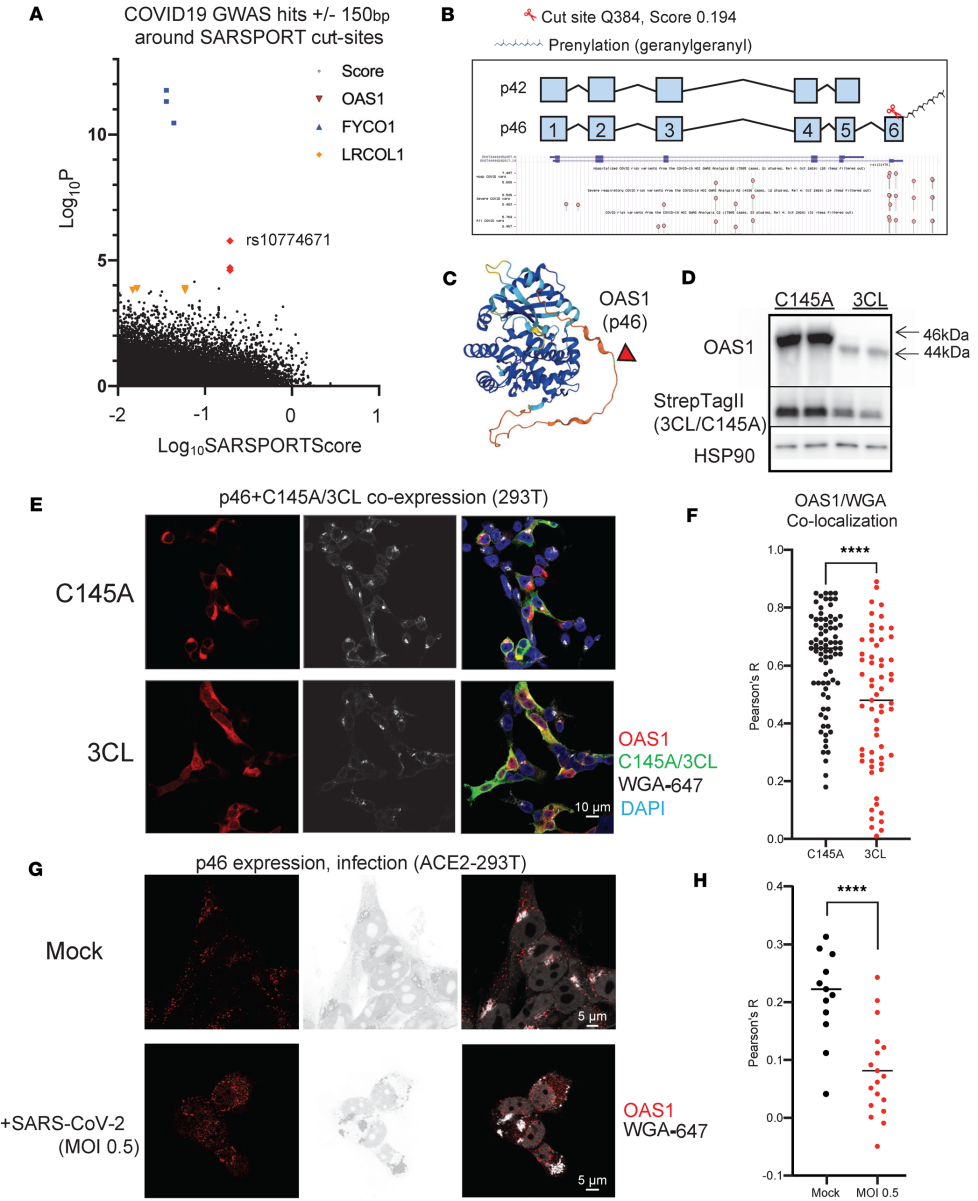
OAS1 degradation and relocalization with 3CLPro expression. (**A**) Identification of COVID-19 GWAS hits adjacent (± 150bp) to genomic locations of SARS-CoV-2 3CLPro cut sites. Log_10_Sarsport score shown on *x* axis, Log_10_*P* value of GWAS hits shown on *y* axis. Highlighted are clusters of high probability SNPs at high-scoring cut sites. (**B**) OAS1 isoforms COVID-19 GWAS SNPs. The p42 (major allele) isoform and p46 (minor alleles) are determine by a single SNP, rs10774671. A high scoring cut-site (Q384) is located 12 aa upstream of the C-terminus of the p46 OAS1 isoform, which encodes a prenylation site. (**C**) AlphaFold structure of full-length p46 OAS1 with predicted cut-site highlighted. (**D**) Western blot of 293T cells overexpressing p46 OAS1 and C145A (catalytically inactive) or active 3CLPro. Full-length (46 kDa) and cleaved product (44 kDa) highlighted. Strep-Tag and HSP90 controls were run on a separate gel from OAS1 staining. (**E**) Immunocytochemistry of 293T cells overexpressing p46 OAS1 and C145A or 3CLPro. Costaining with WGA shown to mark subcellular membrane structures. Scale bar: 10 μm. (**F**) Colocalization quantification of WGA and OAS1 from **E**. In total, 60–80 individual OAS1^+^/3CLPro^+^ or OAS1^+^/C145A^+^ cells were traced and colocalization quantified by Pearson’s R value of linear using the Coloc2 ImageJ plugin (NIH). Comparison statistics of R values quantified by 2-tailed Student’s *t* test. (**G**) Immunocytochemistry of ACE2-293T cells expressing V5 tagged p46 OAS. Costaining of V5 to mark OAS1 with WGA shown to mark subcellular membrane structures. Scale bar: 5 μm. (**H**) Colocalization quantification of WGA and OAS1 from control or COVID infected cells in **G**. Ten to 18 individual cells were traced and colocalization of WGA and OAS1 quantified by Pearson’s R value of linear using the Coloc2 ImageJ plugin. Comparison statistics of R values quantified by 2-tailed Student’s *t* test.

We next sought to identify 3CLPro targets that might be more broadly implicated with COVID-19 disease progression. Recent GWAS have identified a number of host risk loci associated with COVID-19 disease severity (59–63). We cross-referenced the genomic locations of predicted host protein cut-sites to single-nucleotide polymorphisms (SNPs) identified in the COVID-19 Host Genetics Initiative (59) and filtered for significant SNPs (Log *P* > 1) within ± 150 bp of each predicted cut sites, and for sites with high predicted Sarsport1.0 score (>0.01) (Figure 6A and Supplemental Table 6). In particular, we identified clusters of highly significant SNPs around single high-scoring predicted cut sites in *FYCO1, OAS1*, and *LRCOL1*. The predicted cut-site for FYCO1, Q979, has already been identified in a recent screen for 3CLPro cut sites (28), while LRCOL1 is a secreted protein expressed primarily in hepatocytes with mostly unknown function. We thus focused on *OAS1*.

OAS1 is an IFN-inducible innate immune protein upregulated during SARS-CoV-2 infection that senses double-stranded RNA and activates RNase-L (38, 64, 65). There are 2 main protein variants of OAS1, p42 (major) and p46 (minor), which arise due to a single variant splice acceptor site in exon 7, represented by the SNP rs10774671 (G/A; minor allele frequency, 0.39) (66–68). The p46 OAS1 variant encodes a longer C-terminal tail containing a prenylation domain that localizes p46 OAS1 to subcellular membranes (69–71), thus situating OAS1 near viral SARS-CoV-2 dsRNA, which is concentrated within subcellular membrane–derived viral replication structures (72, 73). In contrast, the shorter p42 isoform of OAS1 lacks this C-terminal tail and prenylation site and is primarily cytosolic. Overexpression of p46, but not p42, has been shown to be protective against SARS-CoV-2 infection of cells in vitro (69, 70), and the subcellular localization of p46 drives the protective effect of rs10774671. One of the highest-scoring 3CLPro cut-sites identified by SARSPORT was a C-terminal glutamine (Q384, score 0.194) in the p46 isoform of OAS1 (Figure 6, B and C), which is adjacent to 3 highly significant GWAS SNPs (Log *P* = 4.6–5.7). Q384 lies 12 aa upstream of the critical prenylation site. Thus, we hypothesized that cleavage by 3CLPro may remove the prenylation site and relocalize the protective p46 OAS1 variant from subcellular membranes to the cytosol, thus rendering it unable to detect SARS-CoV-2 dsRNA within membrane-derived viral replication sites. We first confirmed that OAS1 can be cleaved by 3CLPro in vitro. As above, we coexpressed p46 with inactive (C145A) or active 3CLPro for 24 hours. Cleavage was assayed by Western blotting using an antibody that recognizes the OAS1 N-terminus, revealing a near-complete shift in band size of the expected mass (~46 kDa to 44 kDa) (Figure 6D). Immunocytochemistry of p46 OAS1 showed subcellular membrane localization in cells coexpressing inactive, C145A, consistent with the reported localization of OAS1 (69–71). In contrast, the expression of OAS1 in cells expressing active 3CLPro was diffuse and appeared cytosolic (Figure 6E). Quantification (Pearson’s Correlation Coefficient) confirmed a significant decrease in colocalization of p46 OAS1 with subcellular membranes, identified with wheat-germ agglutinin (WGA) (Figure 6F). To validate that this relocalization occurs during infection, we expressed N-terminal V5-tagged p46 isoform of OAS1 in ACE2-expressing 293T cells (ACE2-293T), infected the cells with SARS-CoV-2 at an MOI 0.5, and assessed membrane localization of OAS1 with WGA. As observed with 3CLPro expression (Figure 6, E and F), we saw a significant decrease in membrane localization of OAS1 and its accumulation in the cytosol after SARS-CoV-2 infection (Figure 6, G and H). We thus conclude that 3CLPro cleaves OAS1 at Q384, removing its prenylation site and releasing it from cellular membranes to the cytosol.

## Discussion

We leveraged here experimental data and genome-wide secondary structure analyses to develop a reliable computational algorithm, Sarsport1.0, that predicts endogenous nonviral cleavage targets by the 3CLPro SARS-CoV-2 protease across the human proteome. We validated the precision of the algorithm by confirming predicted cleavage sites, using both biochemical and cell culture approaches. The algorithm is specific: all 9 predicted host proteins that we chose to test experimentally confirmed cleavage by 3CLPro. The algorithm is also precise, accurately correlating scores with the known K_m_/K_cat_ values for the cut sites in the single viral polypeptide. The single exception to this correlation, the NSP9/10 site, is also the only site lying within a predicted β-sheet, which likely hinders protease access. The particularly high score of the NSP9/10 site (score 1.31) may have evolved to overcome this more inaccessible higher order structure.

Sarsport1.0 is highly sensitive, accurately predicting high cut scores in nearly all previously experimentally identified 3CLPro sites (25–28). In addition, thousands of sites are predicted to be cleaved by 3CLPro. Various reasons likely explain why our computational algorithm is more sensitive than prior experimental approaches: (a) experimental paradigms are limited to detecting the proteins expressed in the chosen cell system; (b) proteomic approaches rely on the ability to detect new protein fragments by mass spectrometry, a relatively insensitive method; and (c) proteomic approaches also require the physical presence of cleaved products, but as shown here, these fragments are often rapidly degraded after cleavage. Our computational algorithm overcomes these limitations and provides a comprehensive atlas of the putative SARS-CoV-2 3CLPro degradome.

Our method enabled the identification of a mechanism of SARS-CoV-2 viral defense against host innate immune response: the C-terminal cleavage by 3CLPro of the innate immune protein OAS1. The OAS family of enzymes are a critical component of the OAS-RNaseL innate immune response pathway. Expression of OAS proteins (OAS1/2/3) is induced by viral infection or IFN signaling, and, upon directly recognizing dsRNA, OAS proteins activate RNase-L to degrade viral dsRNA (64, 74). One of the most significant SNPs associated with disease severity in COVID-19 GWAS is a splice acceptor site in OAS1, rs10774671, which determines the expression of either 42 kDa (major) or 46 kDa (minor) isoform of OAS1. The longer, p46 OAS1 isoform has been shown to be protective against severe outcomes of COVID-19 due to a prenylation site at the C-terminus, which localizes OAS1 to subcellular membranes, while the cytosolic p42 OAS1 isoform is not protective. Thus, the highly efficient cleavage by 3CLPro of OAS1 at Q384, just N-terminal to the prenylation site in p46, relocalizes p46 OAS1 away from subcellular membranes and suppresses antiviral activity, somewhat akin to converting the protective p46 isoform to the nonprotective p42 isoform. Supporting this hypothesis, we show that expression of 3CLPro or infection with SARS-CoV2 both drive relocalization of p46 OAS1 from the membrane to the cytosol. The latter was also apparent when examining supplemental data from a prior study (70). Thus we conclude that cleavage of OAS1 by 3CLPro likely acts as a mechanism to neutralize OAS1-mediated innate immunity in patients who express the protective, membrane-localized p46 isoform of OAS1. Interestingly, other coronavirus species such as Middle Eastern Respiratory virus (MERS), express phosphodiesterases that specifically counteract the OAS-RNase-L innate immune pathways by degrading OAS proteins (75–77). SARS-CoV-2, in contrast, does not express phosphodiesterases. Thus, cleavage of OAS1 by 3CLPro may serve as a SARS-CoV-2–specific mechanism of antagonizing the OAS-RNase-L defense pathway, leading to infection even in patients expressing the protective p46 isoform of OAS1.

Our studies with Sarsport1.0 also led us to the identification of the giant sarcomeric protein obscurin as a unique sarcomeric target of 3CLPro, while other sarcomeric proteins remain intact. Ectopic expression of 3CLPro in iPSC-CMs caused dramatic sarcomeric disorganization in a stereotypical fashion similar to that observed with SARS-CoV2 infection (38, 44, 45). Obscurin is a prominent structural component of the sarcomere with critical roles in the assembly, stabilization, and lateral alignment of myofibrils (51). More than 20 missense, frameshift, and splicing variants have been identified in *OBSCN* that are linked to hypertrophic cardiomyopathy (HCM), dilated cardiomyopathy (DCM), arrhythmogenic right ventricular cardiomyopathy (ARVC), and left ventricular noncompaction (LVNC) in humans (78, 79). Many of these mutations are predicted to act through loss-of-function mechanisms due to frameshift-mediated truncations or through haploinsufficiency associated with reduction in obscurin expression (78, 80). We thus propose that sarcomeric disorganization and dysfunction observed in CMs infected with SARS-CoV-2 infection is likely caused in large part by direct proteolysis and loss of obscurin by 3CLPro. Obscurin is also expressed, albeit to varying degrees, in a variety of other tissues such as lungs, kidneys, and brain (54), where its cleavage by 3CLPro may also contribute to severe pathologies due to SARS-CoV-2 infection.

Cardiac complications of COVID-19 have been well documented, and plasma troponin levels are highly predictive of morbidity and mortality after SARS-CoV2 infection (81–83). However, there is limited evidence showing whether the pathology observed in the heart is due to a direct cytotoxic effect of the virus versus secondary to the systemic inflammation. Some evidence of direct infection by SARS-CoV-2 in the human heart has been reported, suggesting that a direct effect is possible (84, 85), although other postmortem studies have not detected infected CMs in patients with COVID-19 (86–88). Thus, the implications of our findings in hPSC-CMs on human cardiac disease should be interpreted cautiously. We used these CM studies primarily as molecular and structural validation of our predictive algorithm, and to demonstrate its utility in generating hypotheses.

The SARS-CoV-2 3CLPro is the target of Paxlovid (nirmatrelvir/ritonavir tablets), an approved therapeutic for the treatment of COVID-19. Since its approval, a retrospective analysis of US patients with COVID-19 has shown that those prescribed Paxlovid within 5 days of diagnosis had a 51% lower hospitalization rate within 30 days, including within vaccinated or previously infected patients (89). Similar results were observed in nirmatrelvir/ritonavir studies around the world (90–93), highlighting the essential role of 3CLPro in disease pathology. Our data suggest that these remarkable benefits of 3CLPro inhibition may accrue from effects beyond suppression of viral replication. For example, infected cells that do not sustain viral replication may nevertheless experience substantial cellular damage from 3CLPro activity on the host proteome. Similarly, as expression of 3CLPro is one of the earliest events in the viral life cycle, cleavage of host proteins such as the OAS1 may contribute to suppression of cellular defenses against infection. Indeed, it will be of interest to determine if nirmatrelvir/ritonavir has a more pronounced effect on patients carrying the p46 allele, versus the p42 allele. The effects of 3CLPro, both during and after viral replication, may also contribute to persistent symptoms, as observed with the long-COVID syndrome. While current studies show that an extended course of nirmatrelvir/ritonavir after acute infection has no to limited effect on long-COVID patients (94–96), there is evidence suggesting that a short course of nirmatrelvir/ritonavir during infection may have a protective effect against developing some symptoms of long-COVID such as brain fog and chest pain/tightness (97). The remarkable benefits of 3CLPro inhibition in patients with COVID-19 underscores the need to further understand the effect of 3CLPro on the host proteome, which will be substantially aided by our predictive algorithm.

In summary, we provide a validated, computationally derived, comprehensive atlas of the putative 3CLPro degradome, overcoming limitations in sensitivity inherent to experimental approaches. In addition, we show that, unlike existing methods, our scoring metric is reflective of relative cleavage efficiency. Our findings demonstrate the power of Sarsport1.0 as a hypothesis-generating tool that can be combined with other large unbiased datasets to uncover virus-host interactions mediated by 3CLPro, the target of highly efficacious therapy against COVID-19. Validating our method, we rediscovered almost all known host protein targets. We identified and validated multiple cleavage sites in the giant sarcomeric protein, obscurin, and demonstrated that 3CLPro cleavage leads in degradation and sarcomere breakdown. And we discovered a clinically relevant cleavage site in the protective p46 isoform of OAS1 that alters its localization to that of the nonprotective, p42 isoform. These results demonstrate how 3CLPro plays a direct role in antagonizing the host cell machinery during early infection. In light of the structural conservation of the 3CL protease, uncovering host targets has implications for understanding outbreaks of both SARS-CoV-2 variants and other coronavirus species in the future.

## Methods

### Sex as a biological variable.

As no human or animal models were used, sex was not considered as a biological variable.

### Bioinformatic prediction of 3CLPro cleavage sites.

In brief, P1 positions (Q, M, or H) were first identified across the human proteome. In total, 8 aa peptides were generated centered at this position, corresponding to P5–P3′ positions in the 3CLPro consensus sequence. Scores were generated by multiplying the relative efficiency values published by Chuck et al. (34) for the SARS-COV 3CLPro. All sites with a score > 0 (i.e., those that did not have an “ND” in any of the positions from P5–P3′) were captured. All captured scores are available under Supplemental Table 1.

### In vitro cleavage assays.

In vitro cleavage assays were performed with purified 3CLPro protein and assay buffer from BPS Bioscience (unconjugated: catalog 100823; MBP-conjugated: catalog 79955). Protease was added at 1 μM concentration, and recombinant protein targets at an approximate ratio of 2 μg target/1 μg 3CLProtein a 50 μL reaction for 1 hour. Recombinant proteins were purchased commercially: NOTCH1 (Origene, catalog TP762041), CDH6 (ACROBiosystem, catalog CA6-H5229), and CDH20 (R&D, catalog 5604-CA-050). Purified human α-thrombin was purchased from Haematologic Technologies (catalog 50-883-435).

### Cell culture.

For 3CLPro overexpression assays, hIPSC ventricular CMs were purchased from Ncardia and cultured according to manufacturer’s instructions. Cells were plated at ~150,000 cells/24-well on fibronectin coated (Sigma, F1131), glass bottomed nano-patterned plates or coverslips (CuriBio, ANFS-0024). Following 4 days of maturation, cells were transduced with adenovirus (Vector Biolabs) to induce expression of StrepII-tagged 3CLPro or catalytically inactive C145A, and lysates were collected at 48 or 72 hours in Thiourea buffer (TU) (see Lysate preparation and Western blotting, below). For MG132 experiments, 1 μM MG132 (Sigma, M8699) was added 24 hours after adenoviral delivery.

For 293T overexpression assays, commercially available 293T (ATCC, catalog CRL 3216) or ACE2-293T (Takara, catalog 631289) were cultured according to manufacturer’s protocol. Transient expression of p46 OAS1, StrepII-tagged 3CL pro, or C145A was induced with transfection of plasmid DNA with Lipofectamine 3000. ACE2-293T cell lines stably expressing V5-tagged p46 OAS1 were generated by third-generation lentivirus delivery followed by neomycin selection. All cells were grown in DMEM+10% FBS media containing 1% Pen/Strep (Gibco, 58570379).

### Human induced-pluripotent stem cells culture and differentiation.

Human induced-pluripotent stem cells (WTC11c hiPSCs, gifted by Bruce Conklin, Gladstone Institutes, San Francisco, California, USA) were maintained in complete mTeSR Plus (Stem Cell Technologies) and cultured on Matrigel-coated dished at 0.17 mg/mL (Corning). WTC hiPSCs were passaged as small clumps for maintenance or single cell-like suspension for cardiac differentiation using Versene (Gibco) and mTeSR Plus supplemented with 10 μM Y-27632 (Tocris). Cardiac differentiation was performed as previously described (PMID: 33657418) (45). Briefly, WTC hiPSCs were seeded at 1,000 cells/cm^2^ using mTeSR1 Plus and 10 μM Y-27632 on Matrigel-coated dishes. After 24 hours, media were replaced with mTeSR Plus supplemented with 1 μM Chiron 99021 (Cayman) to prime the cells for differentiation. Mesoderm induction (day 0) was performed with 3 μM Chiron 99021 in RPMI-1640 media (ThermoFisher) supplemented with 500 μg/mL BSA (Sigma-Aldrich) and 213 μg/mL ascorbic acid (Sigma-Aldrich), named RBA media. After 48 hours (day 2), cells were treated with RBA media supplemented with 2 μM WNT-C59 (Selleckchem). On day 4, media were change with RBA only, and cells were incubated for an additional 48 hours. From day 6 until day 13, hiPSC-CMs were maintained in RPMI-1640 supplemented with B-27 supplement (ThermoFisher). Heat-shock was performed at 42°C for 30 minutes and on day 14, hiPSC-CMs were dissociated using 0.5% Trypsin (Gibco) and cryopreserved in Cryostore CS10 (Sigma).

### HiPSC-CM and ACE2-293T infections.

For CM experiments, before removing samples from BSL-3 containment, samples were inactivated by Thiourea buffer or 4% paraformaldehyde, and the absence of viable SARS-CoV-2 was confirmed for each sample by plaque assays described in the next section.

Frozen hiPSC-CMs were thawed in RPMI 1640 supplemented with B27 supplements, 10 μM Y-27632, and 5% FBS. After 24 hours, media were replaced with RPMI 1640 supplemented with B27 supplements only. A total of 200,000 hiPSC-CMs were seeded 3 days after thawing in Matrigel-coated 24-wells plate using RPMI 1640 supplemented with B27 supplements, 10 μM Y-27632, and 5% FBS. Media were changed after 24 hours, and infection was performed as previously described (PMID: 33657418) (45). Briefly, hiPSC-CMs were quickly washed with DPBS and incubated with SARS-CoV-2 at 5 MOI diluted in DMEM only (Gibco) for 1 hour at 37°C. Mock-control hiPSC-CMs were treated with DMEM only. Media were then replaced with RPMI 1640 supplemented with B27 supplements, and samples were collected at 48 hours after infection.

ACE2-293T infection experiments were performed in the Bio-safety Level 3 Facility at the University of Pennsylvania. Cells were inoculated for 1 hour at 37°C with live virus (SARS-CoV-2, Washington Strain) diluted at an MOI of 0.5, washed 3X with PBS, and incubated for 24 hours with DMEM+10% FBS. Virus was inactivated and cells fixed in 4% paraformaldehyde before proceeding with immunofluorescence staining.

### SARS-CoV-2 preparation and titer.

SARS-Related Coronavirus 2, Isolate USA-WA1/2020 (SARS-CoV-2) was obtained from BEI Resources (NR-52281). Virus propagation and titer was performed in VERO cells (USAMRIID) as described in PMID: 33657418 (45). Briefly, VERO cells were maintained in DMEM supplemented with 10% FBS, 100 U/mL penicillin, and 100 U/mL streptomycin and incubated with either 0.1 MOI (virus propagation) or serial dilution of conditioned media (titer) for 1 hour at 37°C in DMEM only media for viral absorption. For viral propagation, conditioned media were harvested and aliquots were store in –80°C. For titer, 10-fold serial dilutions of conditioned media (either from VERO cells or hiPSC-CMs) were incubated on VERO cells for 1 hour at 37°C. A 1:1 mixture of cellulose suspension (Sigma) and DMEM containing 4% heat-inactivated FBS, L-glutamine, 1X antibiotic-antimycotic (Gibco), and 220 mg/mL sodium pyruvate was layered on top of the cells and incubated at 37°C for 48 hours. Cellulose layer was then removed and cells were stained with 10% paraformaldehyde and stained with 0.5% crystal violet solution in 20% ethanol. Plaques were counted, and the virus titer in the original sample was assessed as plaque-formation unit per mL (PFU/mL).

### Lysate preparation and Western blotting.

For Western blotting of 293T cell lysates, traditional RIPA buffer was used to lyse cells. Lysates were normalized for cell concentration by BCA assay, and denatured by addition of Laemmli buffer (Bio-Rad) at 95°C for 5 minutes. Samples were then run on tris-glycine gels, followed by transfer onto PVDF membranes. Samples were blocked for 45 minutes with 5% milk in TBS + 0.1% Tween-20 and stained overnight at 4°C in Pierce protein-free blocking buffer (Thermo Scientific).

For CM lysates, cells at equivalent densities were first lysed in thiourea denaturing buffer (TU buffer: 8M urea, 2M thiourea, 50 mM Tris-HCl, pH 7.5, 3% SDS, 75 mM DTT). Following incubation in TU buffer for 5 minutes, an equivalent volume of 50% glycerol was added to lysates for a final concentration of 4M urea, 1M thiourea, 25 mM Tris HCl, pH 7.5, 1.5% SDS, 25% glycerol, and 37.5 mM DTT and stored at –80°C until Western blotting. Samples were not heated so as to avoid urea decomposition and subsequent carbamylation of proteins from cyanate ions. Ponceau powder was added directly to lysates for visualization. These denaturing, highly reducing conditions were crucial for solubilization of sarcomeric proteins. Lysates were run directly on tris-glycine or tris-acetate gels, transferred to PVDF membrane, and stained as described above.

### Imaging.

Imaging was performed on a Zeiss LSM 710 confocal microscope at 63X. For sarcomere quantification, lengths of 400 sarcomeres were quantified for 10–15 cells acquired over *n* = 2 independent experiments. Image assignments were blinded during quantification using ImageJ.

### Antibodies.

Antibodies used for Western blotting and immunofluorescence are detailed in Table 1.

### Statistics.

Statistics were calculated with Prism9. For all statistics shown, *P ≤ 0.05, **P ≤ 0.01, ***P ≤ 0.001, ****P ≤ 0.00001. Statistical methods used for all data are indicated in the figure legends for each graph (χ 2 goodness of fit, 1-way ANOVA with Holm- Šidák’s multiple-comparison test, Student’s unpaired, 2-tailed *t* test, ordinary 2-way ANOVA with Šidák’s multiple-comparison test, 1-way ANOVA with Dunnett’s test for multiple comparisons, and R values quantified by 2-tailed Student’s *t* test). All bar graphs with error bars display mean ± SD.

### Study approval.

No patients or animal models were used in the study. All experiments using live virus were performed in the Biosafety Level 3 (BSL-3) facility at the University of Washington and the University of Pennsylvania in compliance with the BSL-3 laboratory safety protocols (CDC BMBL 5th ed.) and the recent CDC guidelines for handling SARS-CoV-2.

### Data availability.

Raw data are provided in the Supporting Data Values file and in Supplemental Tables 1–6.

## Author contributions

NY and ZA conceived and led project ideas and direction. SM generated hPSC-CMs and performed SARS-CoV-2 infections in the laboratory of CM. ET generated the implementation of Sarsport1.0 algorithm. IAK and KL assisted with experimental design and execution, and performed analysis of OAS1 infection experiments. QM performed high molecular weight gels and technical expertise regarding solubilization of high molecular weight proteins. GP performed JPRED analysis of high scoring sites. AR, SS, and NN assisted in protein structure and image analysis. AKK and AG contributed technical expertise and intellectual input on obscurin isoforms and function, as well as obscurin antibodies.

## Funding support

This work is the result of NIH funding, in whole or in part, and is subject to the NIH Public Access Policy. Through acceptance of this federal funding, the NIH has been given a right to make the work publicly available in PubMed Central.

- NIH/NHLBI (HL152446; to ZA).
- NIH training grant 5T32AR053461 (to NY).
- AHA Predoctoral Fellowship (KL) NIAMS T32 training grant (AR 53461-12; to QM).
- Post-doctoral fellowship from the Institute for Stem Cells and Regenerative Medicine (University of Washington; to SM).
- R01 HL128362 and R01 HL146868 to CM.
- Robert B. McMillen Foundation and the State of Washington philanthropical support to the UW Institute for Stem Cell and Regenerative Medicine.
- NIH/NIAMS Training Program in Muscle Biology, T32 AR007592 (to AG).
- NIH/NIAMS R01AR077106 (to AKK).

## Supplementary Material

Supplemental data

Unedited blot and gel images

Supplemental table 1

Supplemental table 2

Supplemental table 3

Supplemental table 4

Supplemental table 5

Supplemental table 6

Supporting data values

## Figures and Tables

**Table 1:**
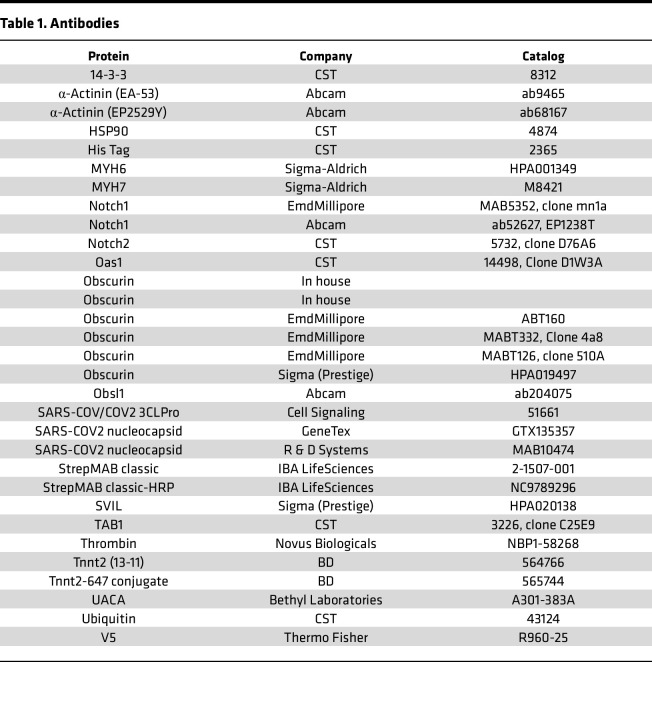
Antibodies

## Version 1 02/23/2026

Electronic publication
