# Supplementary material for: Unbiased cleavage site prediction uncovers viral antagonism of host innate immunity by SARS-CoV-2 3C-like protease
Source: JCI Insight. 2026 Feb 23;11(4):e185739. doi: 10.1172/jci.insight.185739 (PMC12956004; doi:10.1172/jci.insight.185739)
Supplement: Supplemental data [file jciinsight-11-185739-s008.pdf]

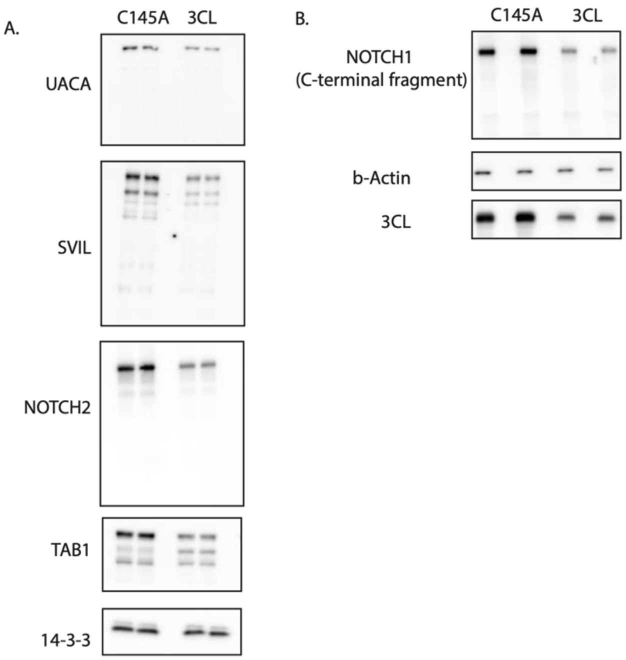

Supplementary Figure 1

A) Western blots of candidate cleavage targets. 48h expression of 3CLPro or C145A in 293T cells.
B) Western blot for the C-terminal cleavage fragment of NOTCH1 following 48h expression in 293T cells.
