# Supplementary material for: Unbiased cleavage site prediction uncovers viral antagonism of host innate immunity by SARS-CoV-2 3C-like protease
Source: JCI Insight. 2026 Feb 23;11(4):e185739. doi: 10.1172/jci.insight.185739 (PMC12956004; doi:10.1172/jci.insight.185739)

Full unedited blots for Figure 2A

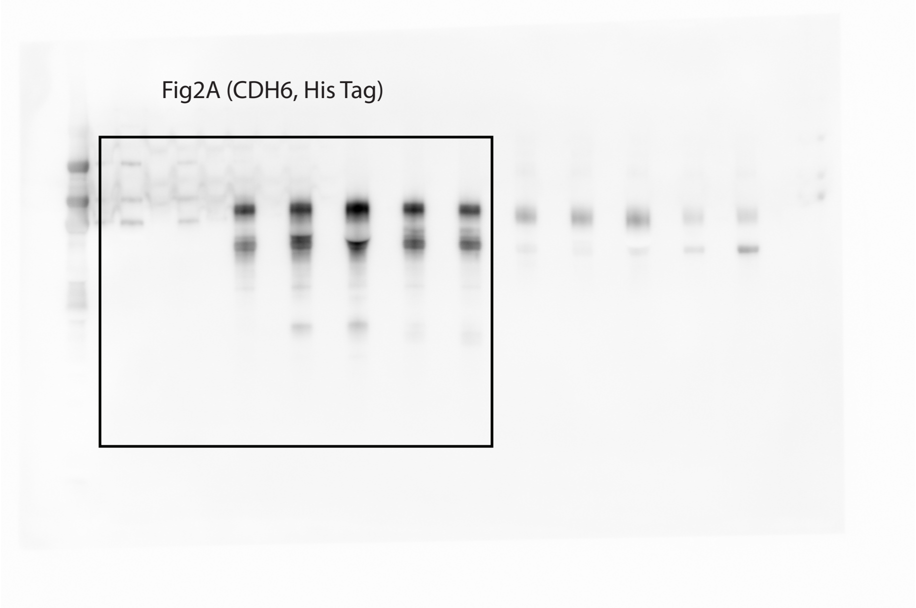

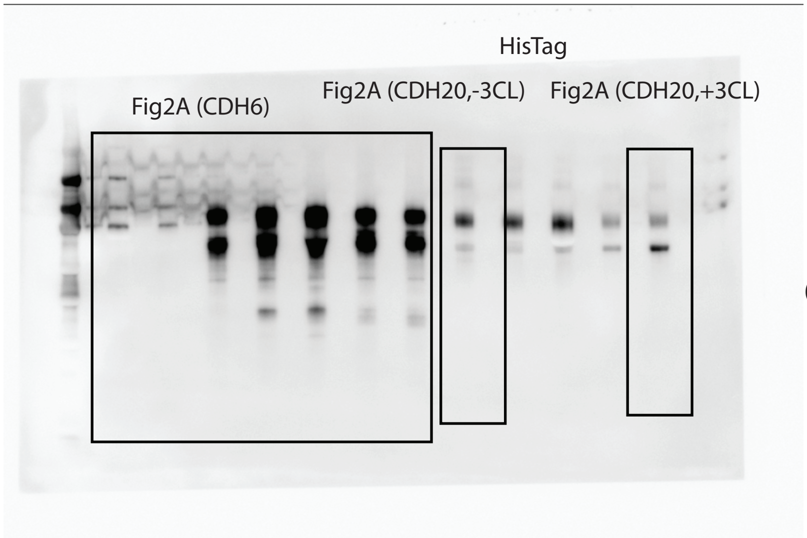

(Previous blot, increased exposure)

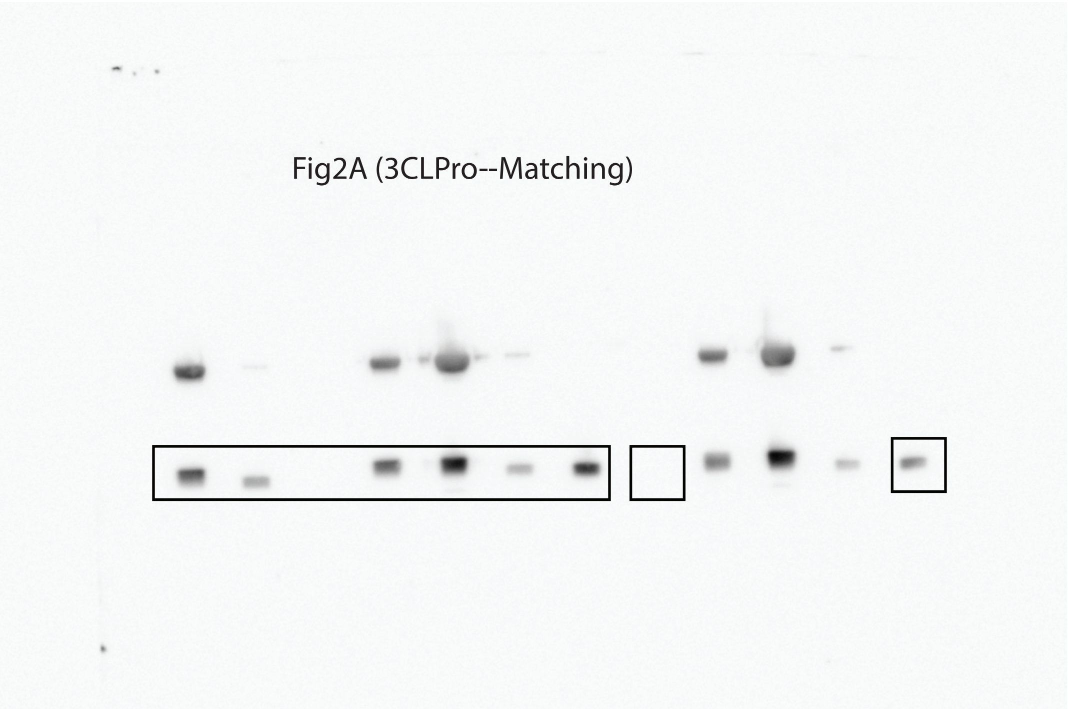

Full unedited blots for Figure 2B-D

Fig2B (IIa)

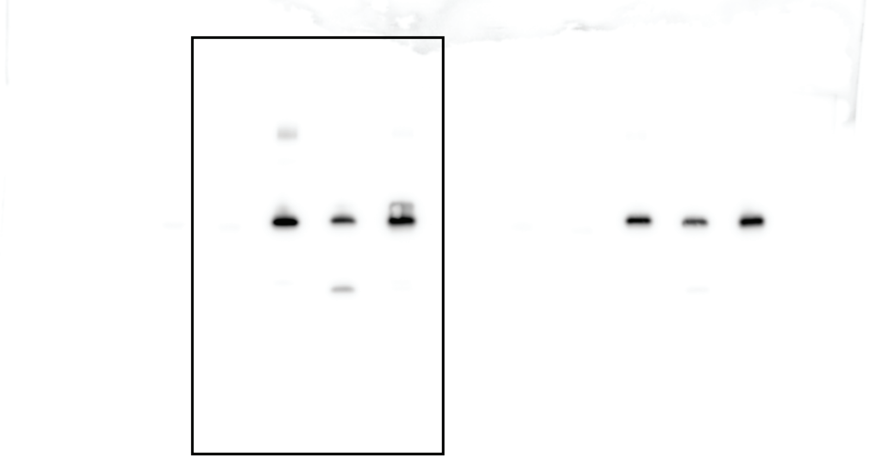

Fig 2C: HisTag

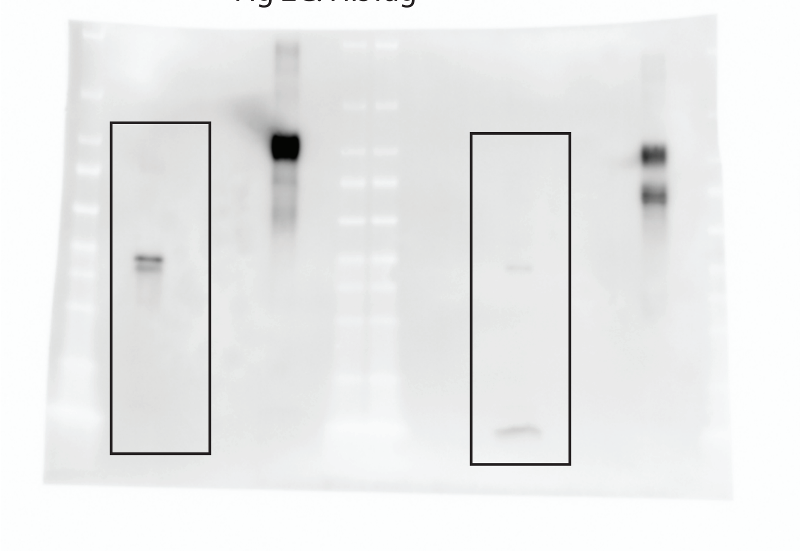

Fig 2C: NOTCH1 (EP1238Y)

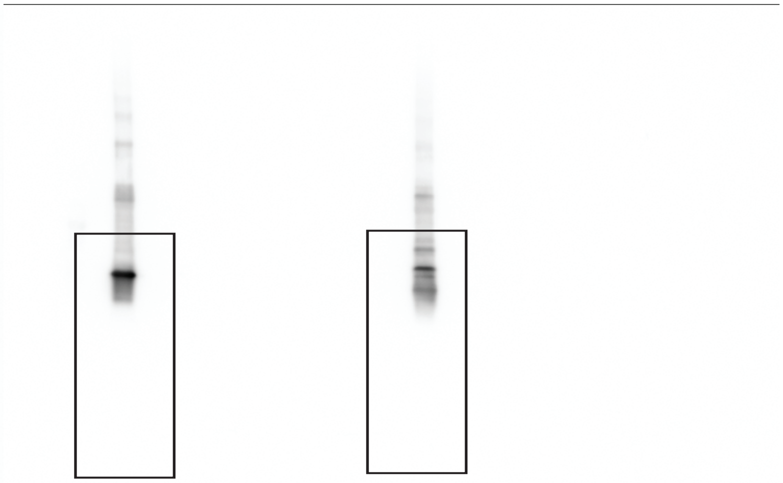

Fig2D(mn1a)

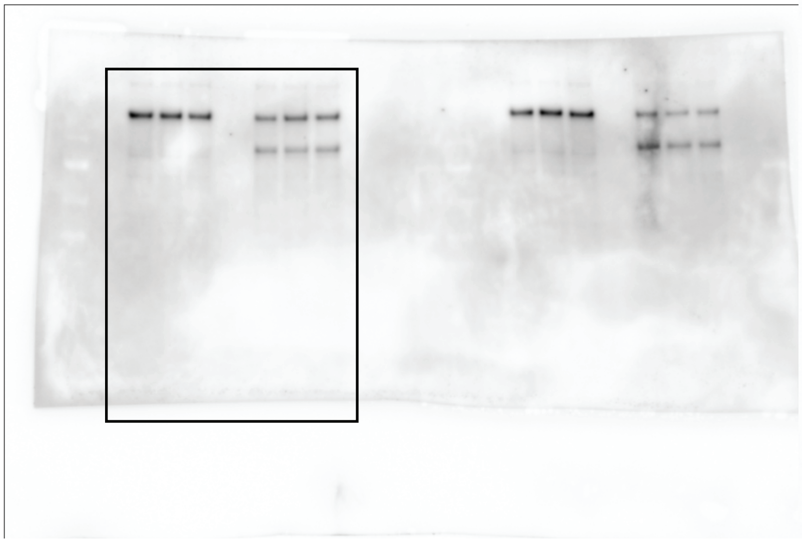

Fig2D(beta-actin)`

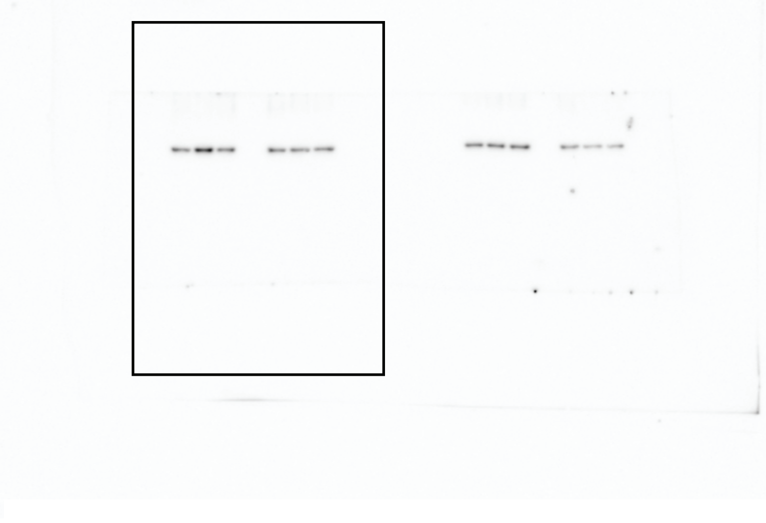

Full unedited blots for Figure 4B

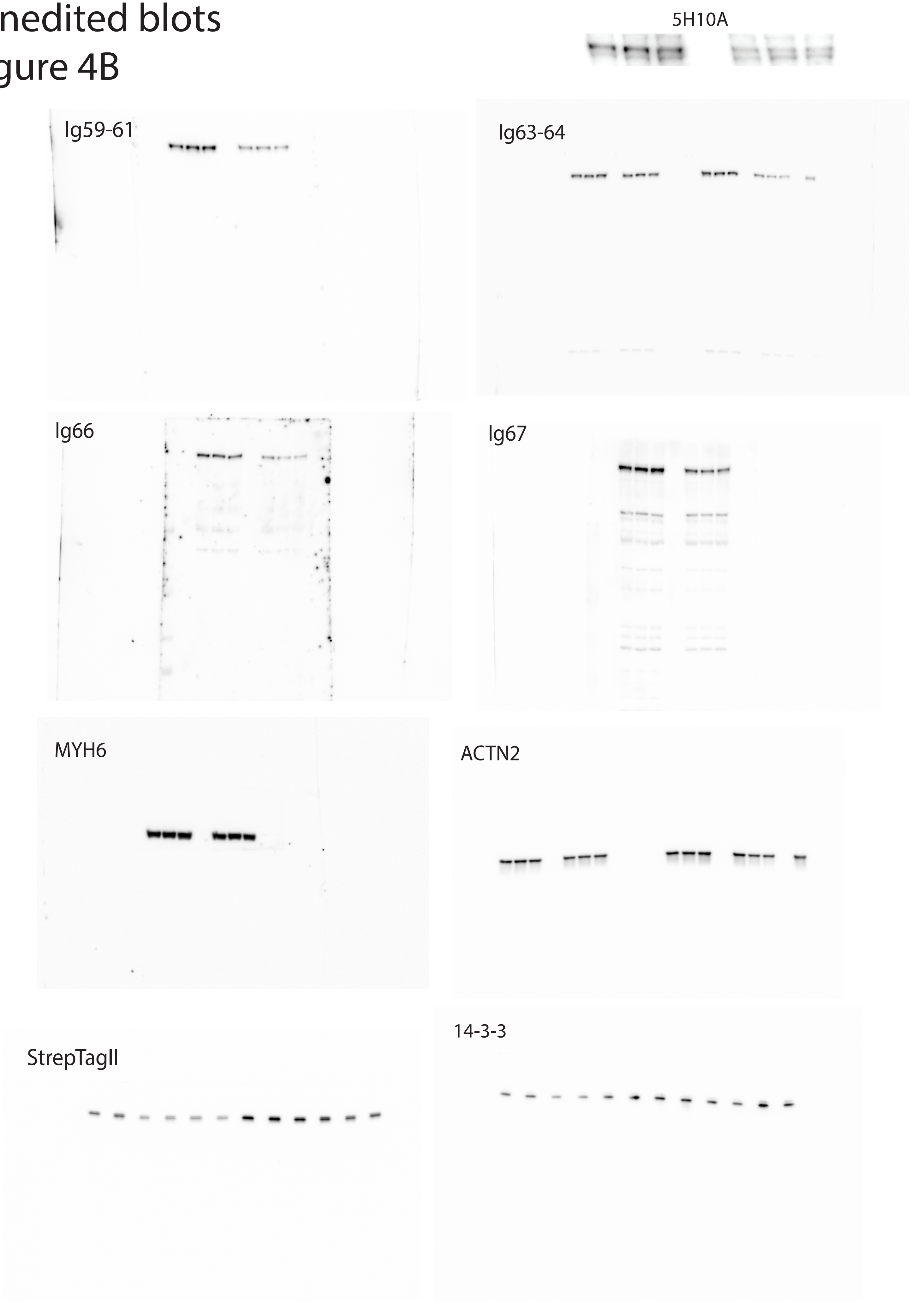

Full unedited blots for Figure 4E

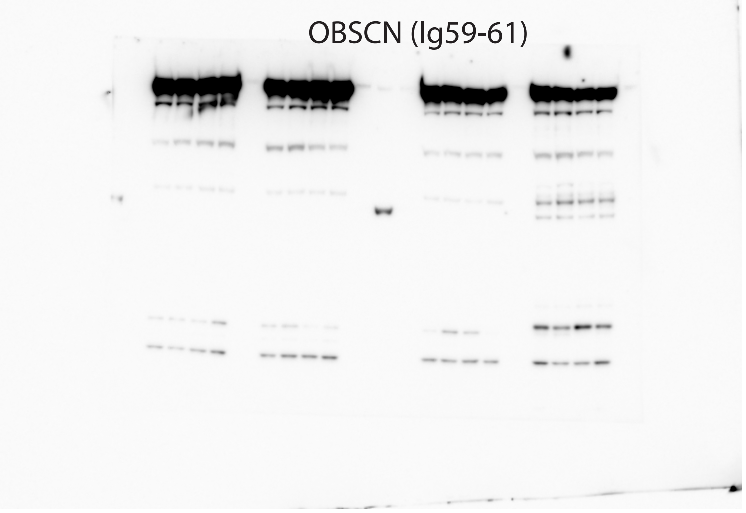

OBSCN (Ig63-64)

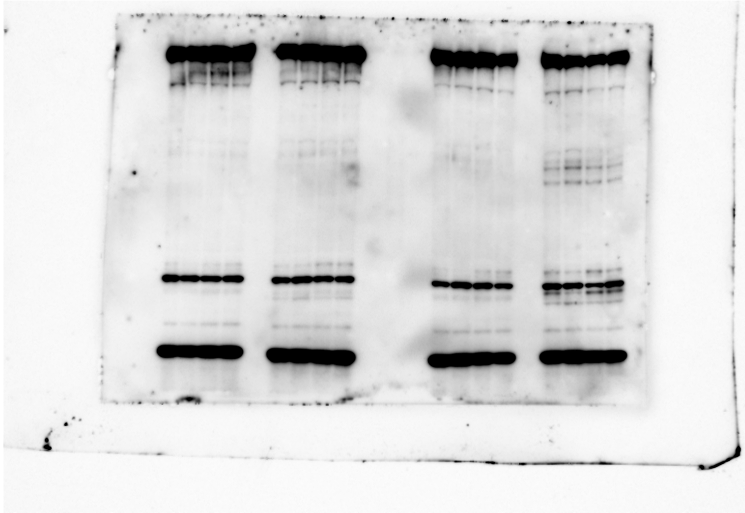

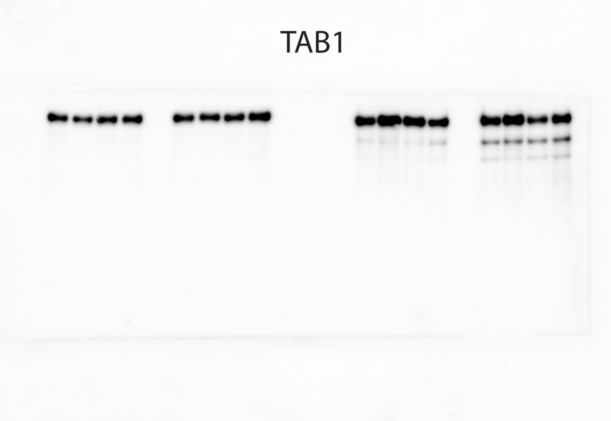

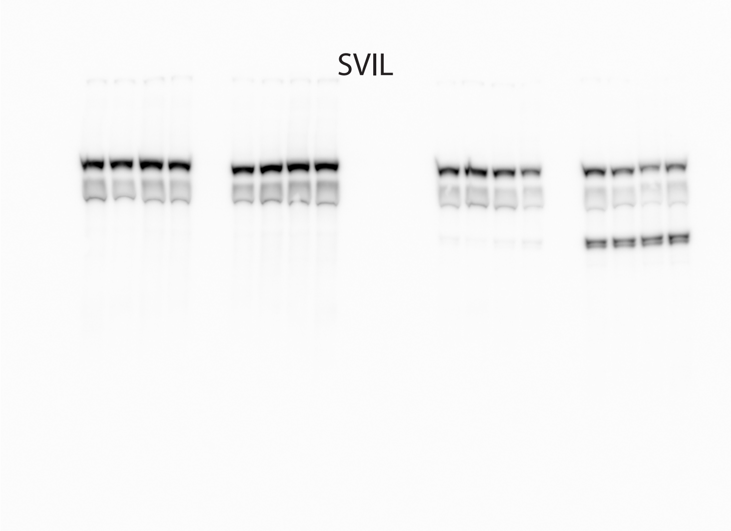

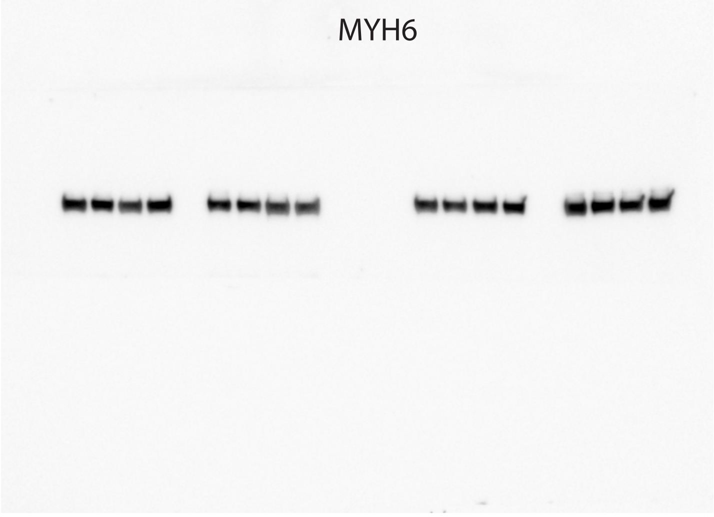

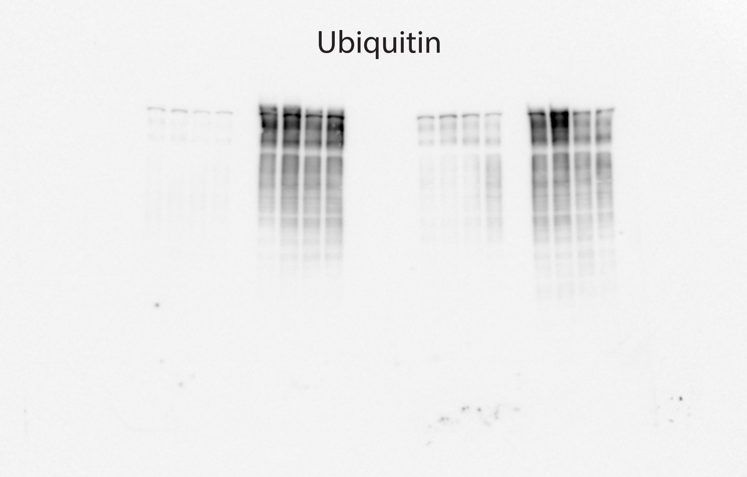

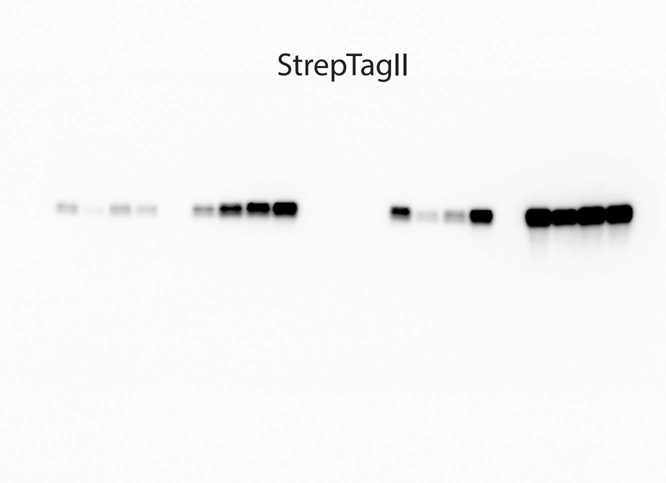

Full unedited blots for Figure 5A

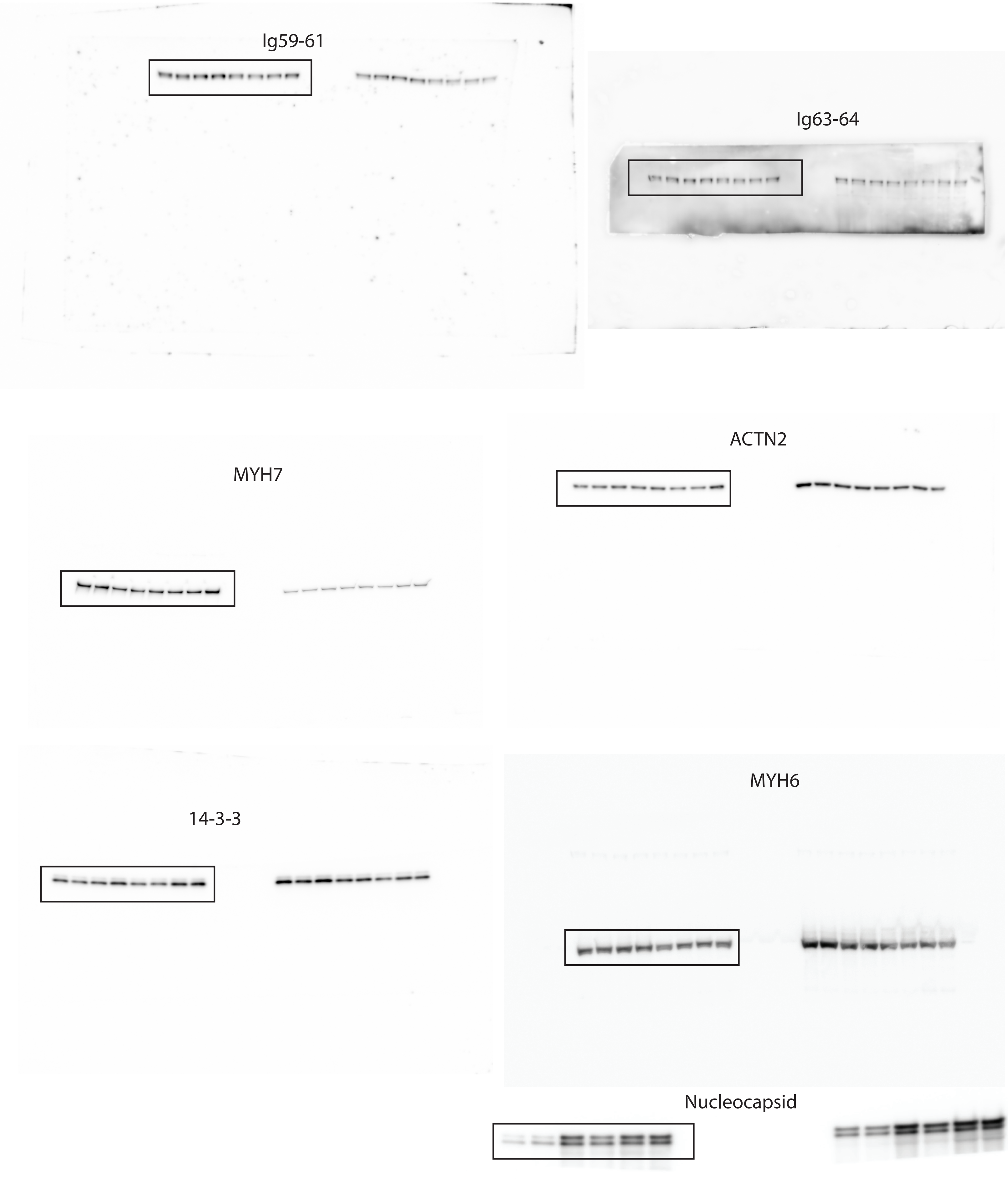

Full unedited blots for Figure 6D

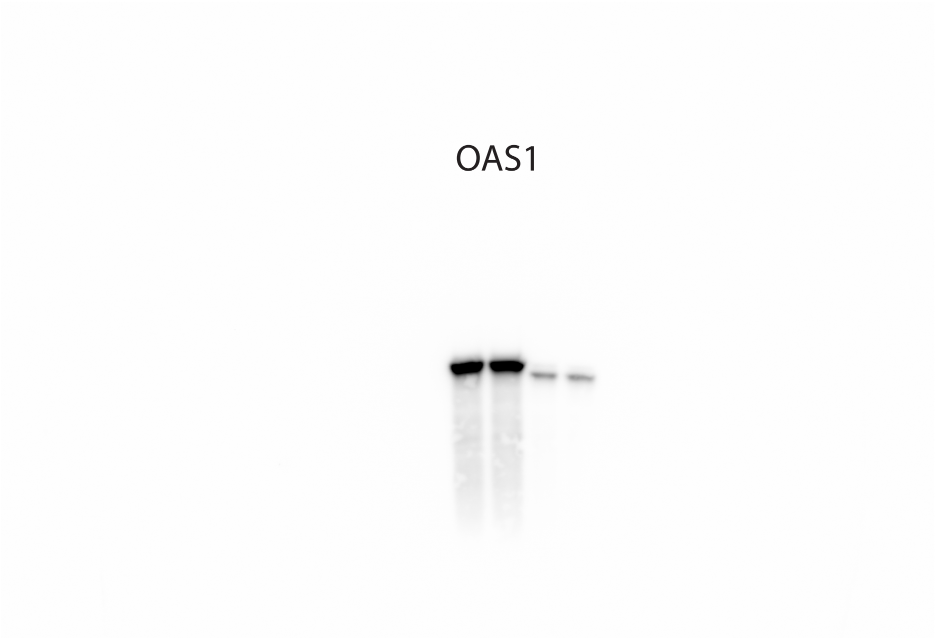

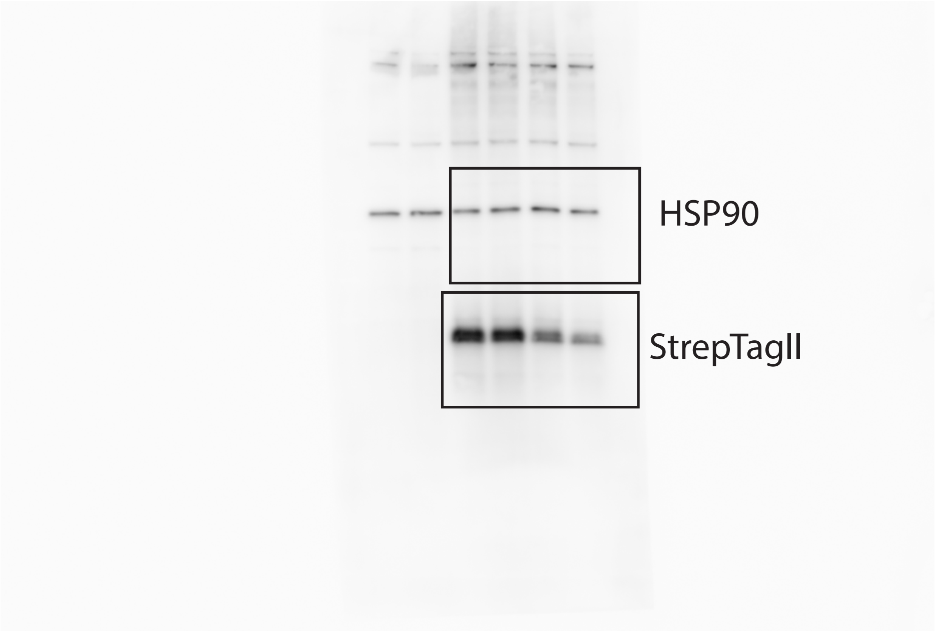

Supplement: Unedited blot and gel images [file jciinsight-11-185739-s009.pdf]
